# From Wet to Protective: Film Formation in Waterborne Coatings

**DOI:** 10.1021/acsami.4c09729

**Published:** 2024-10-21

**Authors:** Abolfazl Arjmandi, Huichao Bi, Stefan Urth Nielsen, Kim Dam-Johansen

**Affiliations:** † CoaST, Department of Chemical and Biochemical Engineering, Technical University of Denmark (DTU), Building 229, Kgs. Lyngby 2800, Denmark; ‡ R&D Department, Hempel A/S, Lundtoftegaardsvej 91, Kgs. Lyngby 2800, Denmark

**Keywords:** waterborne coating, film formation, anticorrosive, latex, coalescing agent

## Abstract

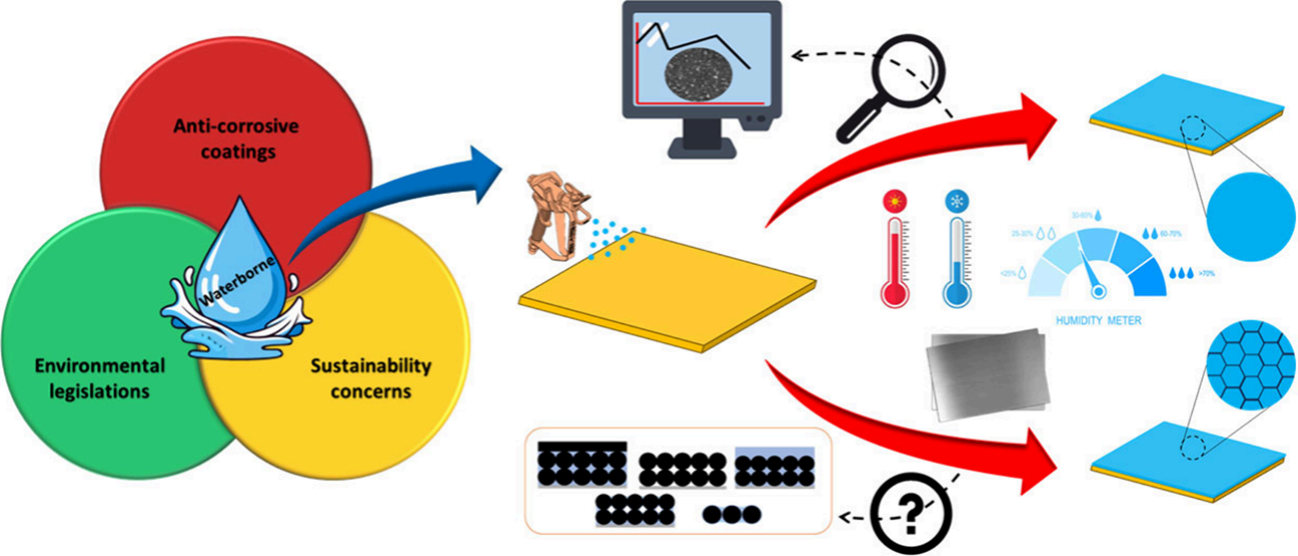

The importance of anticorrosive coatings cannot be overstated, as they play a vital role in safeguarding assets and infrastructure across various industries. Within this context, the emergence of waterborne (WB) coatings stands out for their paramount significance, offering a sustainable alternative to traditional solvent-based (SB) coatings and addressing pressing environmental concerns. Despite their eco-friendliness, the complexity of their film formation mechanism and the lack of understanding present challenges in enhancing the performance of waterborne coatings for corrosion protection. These coatings are created by dispersing polymers in water, which then form a protective film on a substrate. The process involves stages like water evaporation, particle packing, and polymer interdiffusion, often leading to films with defects and inferior protection against extremely corrosive environments compared to SB coatings. This Review scrutinizes the interplay of factors affecting film formation in application, including coalescing agents, environmental factors, and application conditions. A comparative analysis between SB and WB coatings is also featured to shed light on the performance gap under harsh conditions. This Review discusses analytical techniques for studying film formation, aiming to guide future research toward improving WB coatings’ durability and effectiveness. In compiling this collective wisdom, this Review emphasizes the translation of theoretical understanding into practical knowledge, equipping formulators with actionable insights to optimize WB coatings for real-world application and performance.

## 1 Introduction

Corrosion is a universal problem across many industries, causing billions of dollars in damages annually.^1^ Anticorrosive coatings are a cost-effective and efficient solution to mitigate the damage caused by corrosion.^2^ WB coatings provide enhanced sustainability compared to SB coatings through reduced volatile organic compound (VOC) emissions, lower hazardous chemical use, decreased environmental impact, and improved workplace safety. They align with strict environmental regulations, consume less energy, and utilize sustainable raw materials. Their reduced environmental footprint and safer application make them a preferable choice for various industries, supporting the global shift toward sustainability and reduced environmental impact.^3−6^ These coatings are based on water-soluble or water-dispersible polymers, which are dispersed in water using surfactants and stabilizers to form stable dispersions. Upon application, these dispersions coalesce to form a continuous film on the substrate, providing excellent protection against corrosion.

While WB coatings have improved in corrosion resistance over the years, they are compared to SB technologies still less effective overall in providing long-term protection against corrosion in aggressive conditions, such as industrial, high humidity, aggressive atmosphere, coastal and offshore areas with high salinity, temperate and subtropical with high pollution and/or significant chloride effects, and immersion services in marine environments.^7,8^ Waterborne epoxy systems typically incorporate hydrophilic groups or surfactants, which are less effective at preventing the penetration of water, oxygen, and other corrosive ions. This characteristic makes them less resistant to corrosion compared to other systems.^9,10^ It is worth mentioning that even the advantage of WB coatings from the sustainability point of view depends on their performance, as they need to be of at least equal lifetime compared to their SB counterparts to be considered as a more efficient option.^11^ Despite the prevalent adoption of WB anticorrosive coatings, the intricate nature of the film formation process for these coatings remains a subject of ongoing complexity and limited understanding. Achieving a continuous coating film from WB dispersions involves a multistep mechanism, encompassing factors such as water evaporation, particle arrangement, and the diffusion of polymer chains between adjacent particles.^12^ This complexity arises from the composition of WB dispersions, which consist of discrete polymer particles dispersed within an aqueous medium. Consequently, in comparison to films created from solutions where the polymer is entirely dissolved in an organic solvent, films originating from WB dispersions tend to exhibit a higher incidence of imperfections and diminished mechanical as well as water resistance. These characteristics render them at present susceptible to elevated levels of corrosion and exposure to extreme environmental conditions. The factors that affect the film formation process include the type and properties of the polymer, the surfactant and stabilizer used, the coalescence agent, and the application conditions.^13−15^ Coalescence agents, in particular, are crucial cosolvents in the film formation process, as they promote the merging of adjacent polymer particles through decreasing the glass transition temperature (*T*_g_), leading to the formation of a continuous film. The properties of the coalescence agent, such as its molecular weight, concentration, and hydrophilic–lipophilic balance (HLB), can significantly impact the film formation process and the properties of the resulting coating.

Moreover, application conditions, such as temperature, humidity, and substrate preparation, can also significantly affect the film formation process. For instance, low temperatures can hinder the coalescence of polymer particles by being below *T*_g_ and limiting the evaporation of water, leading to the formation of a weak and porous coating.^16^ On the other hand, high temperatures can cause rapid evaporation of the water, leading to the formation of defects, such as pinholes and bubbles, in the coating.^6^ Additionally, the substrate’s cleanliness and surface energy can also impact the film formation process by affecting the wetting and adhesion of the coating.^17^

Over the years, several studies have investigated the effect of coalescence agents and environmental conditions on the film formation mechanism of WB anticorrosive coatings. However, a comprehensive understanding of the interactions between these factors is still lacking. Therefore, this Review aims to analyze the existing research and identify the current state of knowledge regarding the film formation mechanism of WB anticorrosive coatings concerning the environmental and application effects. This Review aims to provide insights into the factors that influence the film formation process, which could aid in the development of more effective and durable WB anticorrosive coatings with application properties that allow them to be used in environment conditions currently not possible.

## 2 Applications and Challenges of WB Coating Technology

### 2.1 Applications

**Figure 1:**
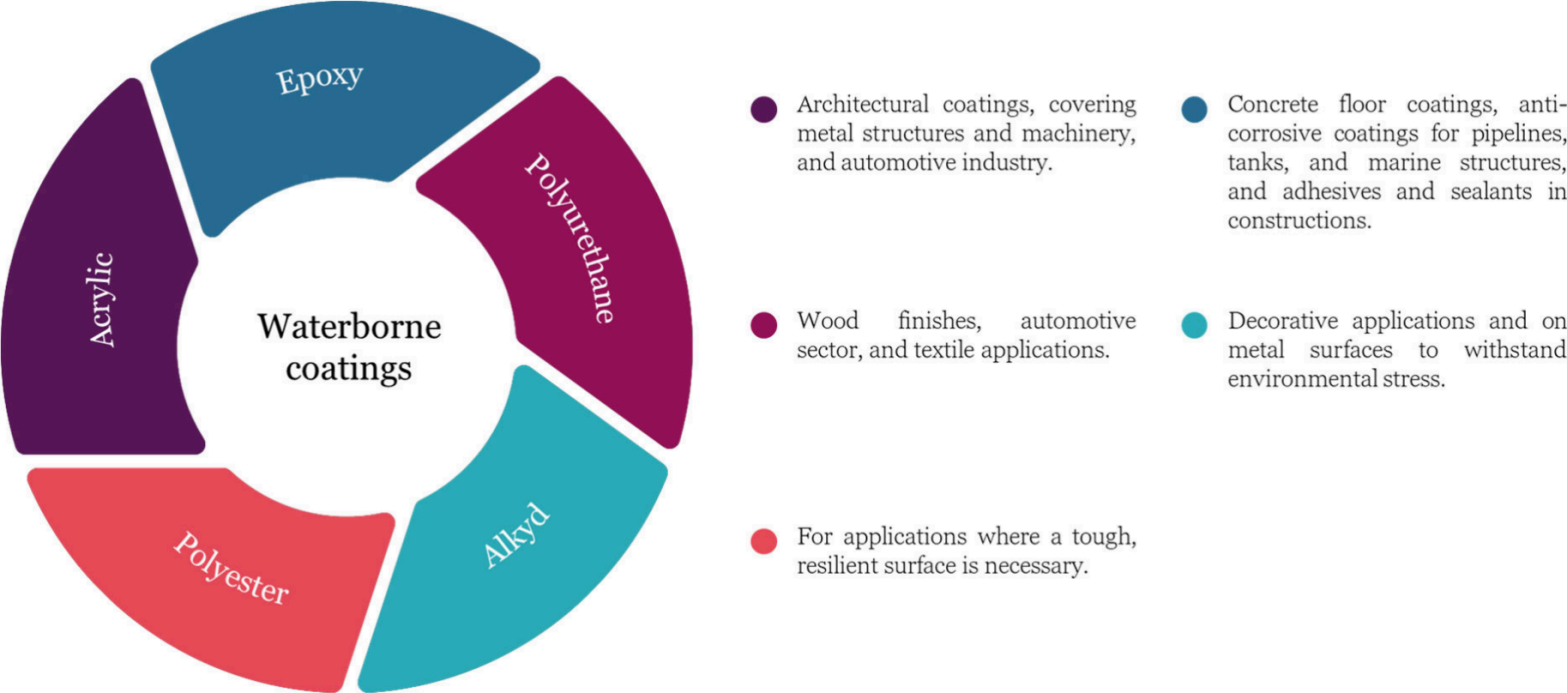
Most common applications of WB coatings in different industries.

WB coatings have become increasingly popular due to their reduced environmental impact and versatility across multiple industries.^18^ A classification of all commercially available WB coatings with application examples for each of them is illustrated in Figure 1. WB acrylic coatings, formulated from acrylic or styrene-acrylic polymers, are widely appreciated for their durability, UV resistance, and quick drying times. These characteristics make them particularly suited for architectural coatings, where they are extensively used in both interior and exterior house paints. Their resistance to weathering and excellent adhesion also makes them ideal for industrial coatings, covering metal structures and machinery, and in the automotive industry for topcoats, ensuring long-lasting color retention and durability. WB epoxy coatings, derived from epoxy resins, offer superior chemical resistance, adhesion, and durability. These properties are particularly valuable in concrete floor coatings, making them a preferred choice for industrial and commercial flooring where high performance is essential. Their protective qualities extend to applications in harsh environments, such as pipelines, tanks, and marine structures, where resistance to corrosion is crucial. Additionally, WB epoxies are used as adhesives and sealants in construction and industrial sectors, where strong bonding and resistance to chemicals are required. WB polyurethane coatings, based on polyurethane dispersions, combine flexibility, abrasion resistance, and chemical resistance. These attributes are highly valued in wood finishes, where they provide a clear, durable finish, making them ideal for furniture and flooring. In the automotive sector, they are employed in refinishing processes, particularly in clear coats and base coats, due to their excellent gloss and durability. Furthermore, these coatings are used in textile applications to impart water resistance, durability, and a soft texture to fabrics. WB alkyd coatings, which involve alkyd resins modified for water dispersibility, are known for their glossy finish and smooth flow characteristics. These coatings are popular in decorative applications, such as interior and exterior house paints, where a shiny finish and strong adhesion are desired. In industrial settings, they are used on metal surfaces, providing a durable, glossy finish that withstands environmental stress. They are also applied in wood coatings, where their ability to penetrate the substrate and deliver a smooth finish is particularly beneficial. Lastly, WB polyester coatings, formulated from polyester resins, are recognized for their hardness, chemical resistance, and durability. These coatings are often utilized in applications where a tough, resilient surface is necessary, although their specific real-world applications can vary depending on the exact formulation and performance requirements. Each type of WB coating is tailored to meet the specific needs of various industries, making them integral to many real-world applications.

### 2.2 Current Challenges

Conventional coatings consist of solvent-dispersed resins, pigments, fillers, and additives, with solvent evaporation and film formation commencing immediately postapplication, crucial for coatings’ complete curing.^19^ However, the evaporation process, involving solvents like toluene and xylene, poses health risks such as cardiac arrhythmia and irritation of the eyes, nose, and throat to individuals and applicators.^20,21^ Recent regulatory measures aim to mitigate the environmental and health impacts of volatile organic compounds (VOCs) by enforcing stricter VOC limits and promoting environmentally friendly practices, including the capture of hazardous vapors and the development of solventless, WB, or high-solids coatings.^18^ WB coatings, utilizing water to disperse resins, offer environmental benefits and are divided into the emulsion, water-dispersible, and water-soluble categories, each with unique characteristics and applications.^22−25^

Although WB coating systems have many advantages over traditional SB coatings, they also have some disadvantages, especially when it comes to high and extreme corrosivity environments. Most of these challenges originate from the diverse components and the complex film formation process in WB coatings, making them more susceptible to environmental factors during application compared to SB coatings.^26^ The ultimate aim of a coating process is to achieve a uniform film formation with strong adhesion to the underlying substrate. Anticorrosive performance of WB coatings is strongly dependent on application and drying conditions, such as temperature and humidity.^27^ WB coatings typically consisted of various components, including water, polymer binders, coalescing agents, pigments, fillers, defoamers, neutralizing agents, rheological additives, surface levelers, preservatives, plasticizers, wetting and dispersion additives, etc.^28^ The inherent challenge lies in the fact that a significant portion of these components exhibits insolubility or partial solubility in water, thereby predisposing the coating formulation to instability and the potential for phase separation. To prevent phase separation, a commonly adopted strategy involves the integration of amphiphilic fragments and components. The problem with this solution is the creation of complex interactions, which renders it challenging to effectively control and adjust the ultimate properties of the coating film.^15^ Moreover, the limited application window of waterborne coatings exemplifies the broader challenge of balancing environmental sustainability with practical application needs. The challenge of balancing a low minimum film formation temperature (MFFT) with high mechanical strength in waterborne coatings arises from the conflicting demands these properties place on the formulation. A low MFFT is crucial for enabling film formation at lower temperatures, particularly in colder climates, but achieving this often involves using softer polymers or plasticizers that can weaken the coating’s mechanical strength. Conversely, to ensure high mechanical strength, which is essential for durability and resistance to wear, harder polymers are typically used, which increases the MFFT and restricts the application window to warmer conditions. Overcoming the film formation dilemma without using coalescing agents has been approached through physical^29,30^ and chemical cross-linking,^31,32^ blends of soft and hard particles, and hybrid polymer latexes with core–shell structures.^33−35^ While cross-linking complicates the chemistry and blends risk phase separation, hybrid latexes with soft core–hard shell designs can improve mechanical strength but may cause cracking due to stress during film formation. Reducing particle size can lower MFFT and improve mechanical properties, but often increases brittleness, presenting a trade-off in performance.

**Figure 2:**
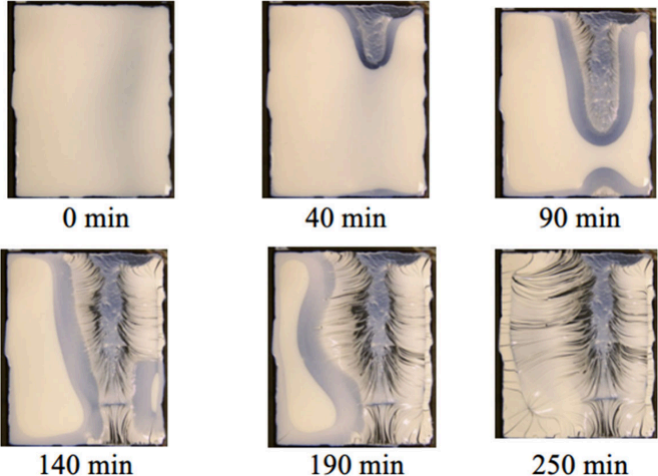
Optical images evidencing the nonuniform horizontal drying of an applied WB coating (200 nm polystyrene particles in water) with 10 wt % solid concentration. Reproduced with permission from ref (36). Copyright 2013 IOP Publishing Ltd.

The complexity of film formation in WB coatings is further highlighted by Routh and colleagues, who demonstrated through visual evidence that the drying process is uneven across the film. Figure 2 illustrates that the edges of the film tend to dry first, resulting in a nonuniform drying pattern.^36^ The initial image, taken at zero minutes, reveals a film in a fully liquid state. As the drying process advances, the film gradually transforms into a solid structure, as evidenced in the image’s top portion captured at 40 min. The milky white fluid area diminishes in size but remains discernible even past the 190 min mark. Simultaneously, the solidified region extends horizontally across the film’s surface, giving rise to what is termed a “horizontal drying front”, signifying the boundary between the fluid and solid phases of the film. The driving force behind this heterogeneous drying process is not discussed in their study. The sample they used in their study is only 10 wt % (making an intense heterogeneous drying) to have clear visual evidence of horizontally heterogeneous drying behavior; however, in other studies using high concentrations (up to 50 wt %), this drying behavior is also proved.^37^ A smaller particle size, a quicker evaporation rate, and a thicker film encourage water to recede from the edges of a WB colloidal film during drying. Conversely, a larger particle size, a slower evaporation rate, and a thinner film result in more uniform drying across the lateral direction.

## 3 Film Formation of WB Coatings

**Figure 3:**
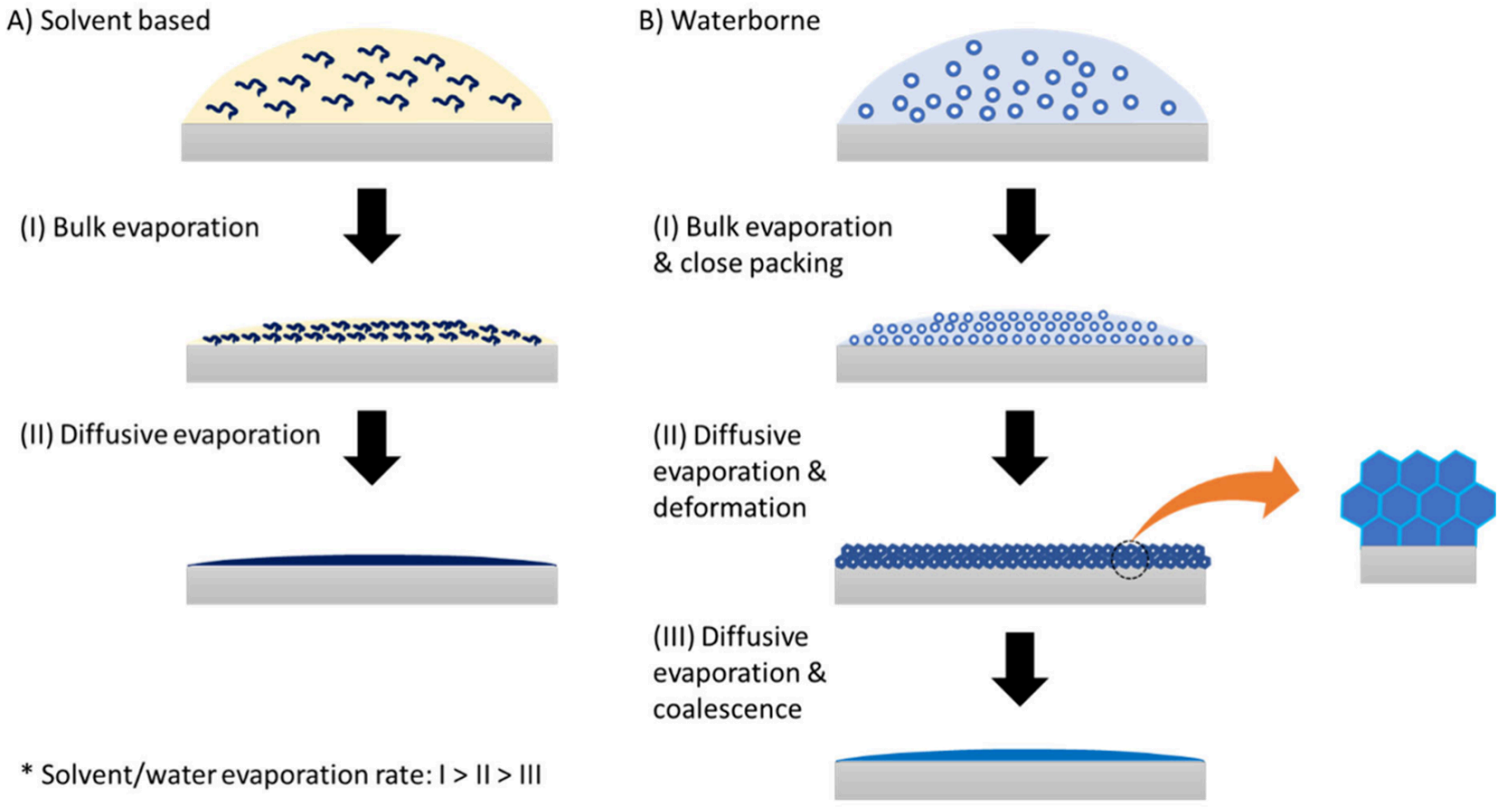
Schematic representation of the film formation stages for different coating systems at a simplified condition: (a) for SB coatings (dissolved polymer) and (b) for WB coatings (dispersion of latex in water).

Extensive research across multiple disciplines has been conducted to understand the mechanisms involved in WB and SB film formation processes. The current understanding of theoretical stages involved during the film formation of SB polymers (a)^38^ and WB polymer emulsions (b)^39^ is illustrated in Figure 3. In SB coatings (Figure 3a), the drying process can be divided into bulk evaporation (I) and diffusive evaporation (II). The freshly applied film leads to the formation of a dense polymer network as the solvent rapidly evaporates by a mass value of approximately 90%. In the second stage, the remaining solvent gradually evaporates over time, with a reduced evaporation rate due to the compact network formed in the first stage. However, high film thickness can lead to problems with entrapped solvents, resulting in defects and incomplete film formation. It is worth noting that the mechanisms illustrated in Figure 3 are simplified processes ignoring the effects of other components, such as pigments, which can affect the whole mechanism through their interactions with the water/solvent and polymer particles.

Conversely, WB films form through a unique procedure comprising three phases, as illustrated in Figure 3b. In the initial state, the dispersed latex particles arrange themselves in a close-packed manner, resulting in a minimal presence of water and additives within the structure (approximately 36 vol % water and cosolvents in an ideal close-packed configuration). Following this, water and other space-filling additives leave the interstices between the latex spheres during the next stages, which is facilitated by capillary pressure. This crucial process is supported by the use of coalescing agents, which temporarily soften the latex, enabling its deformation and allowing the residual water and other additives to evaporate. As a result, a continuous and densely packed polymer film is formed. Achieving a successful film formation necessitates the meticulous completion of all three steps in a sequential manner. This intricate process renders WB coatings significantly more sensitive during film formation compared to their counterparts in SB coatings. There are a wide range of variables to disturb this process, leading to the formation of a defective coating to be ineffective against extremely corrosive environments. The ability for particle deformation and polymer interdiffusion is contingent upon a critical condition: the ambient temperature must be higher than the effective *T*_g_ of the polymer. Only under such circumstances can these processes take place effectively.^40^ A successful particle deformation process is crucial to avoid undesired outcomes such as a powdered polymer or a cracked film. Furthermore, a deficient polymer interdiffusion phase can significantly undermine the mechanical characteristics of the eventual film. In order to adhere to the specifications of organic coatings, polymers with elevated *T*_g_ are frequently employed to prevent common final film issues like tackiness and softness. However, this choice introduces greater complexity to the film formation process. Various approaches have been explored to address this challenge, including the incorporation of coalescing agents,^41^ the use of blended particles,^42^ the implementation of multiphase particles,^43^ or a combination of these strategies.^44^

### 3.1 Close-Packed Structure

**Figure 4:**
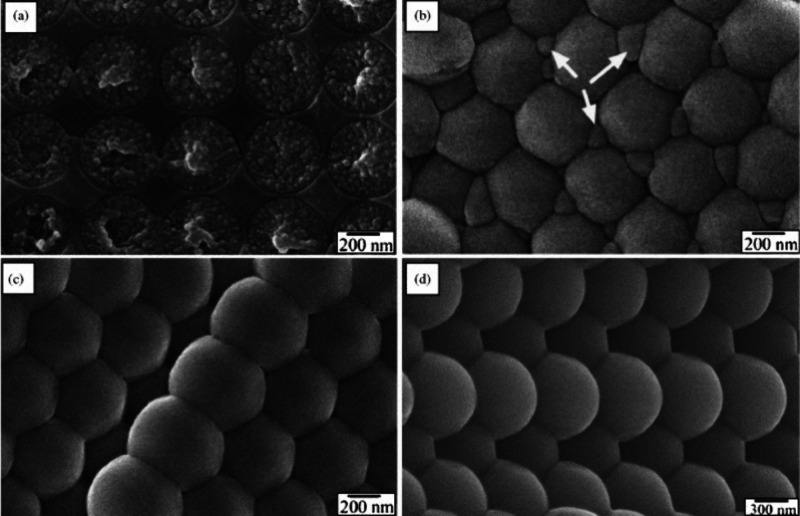
Cryogenic SEM (cryo-SEM) images of latex after the evaporation of water for (a) 3 min, (b) 6 min, (c) 8 min, and (d) 15 min, going from a close-packed (ordered) structure to a deformed structure with distinguishable boundaries. The small features between particles shown by arrows are trapped frozen water. Reproduced with permission from ref (48). Copyright 2005 Elsevier.

The presence of particles in the dispersion causes the wet regions to appear turbid due to light scattering. Under certain conditions, there is a possibility of colloidal crystallization, wherein the particles arrange themselves in an ordered array during slow drying. However, more commonly, the particles tend to accumulate in a random close-packing arrangement, with the volume fraction reaching approximately 0.64 for monosized particles.^45−47^ This indicates that around 64% of the available space is occupied by spherical particles, with the remaining spaces filled with water. Figure 4a provides a visual representation of the close-packed structure of a group of particles. The arrangement of individual particles resembles a mountain range, with the peaks and valleys corresponding to the particle radius, creating a distinctive topography.

### 3.2 Deformation

As the spherical particles in the dispersion come into contact, they tend to fill in the interparticle void space by deforming from their original shape. This deformation can result in the particles retaining their distinct individual identities. During this process, the interparticle voids gradually diminish, leading to a significant change in the optical properties of the particle layer. With the reduction of interparticle voids, the particle layer transitions from being optically turbid to becoming optically transparent. This occurs because the presence of heterogeneities in the refractive index, which previously caused light scattering, is minimized. In many cases, the point at which the film becomes optically transparent is used as a defining characteristic to mark the completion of film formation. This milestone signifies the attainment of a more cohesive and densely packed structure, where light can pass through the film without significant scattering. The onset of transparency in the film serves as an important indicator of the evolution of its optical properties and signifies the achievement of a critical stage in the film formation process.

**Figure 5:**
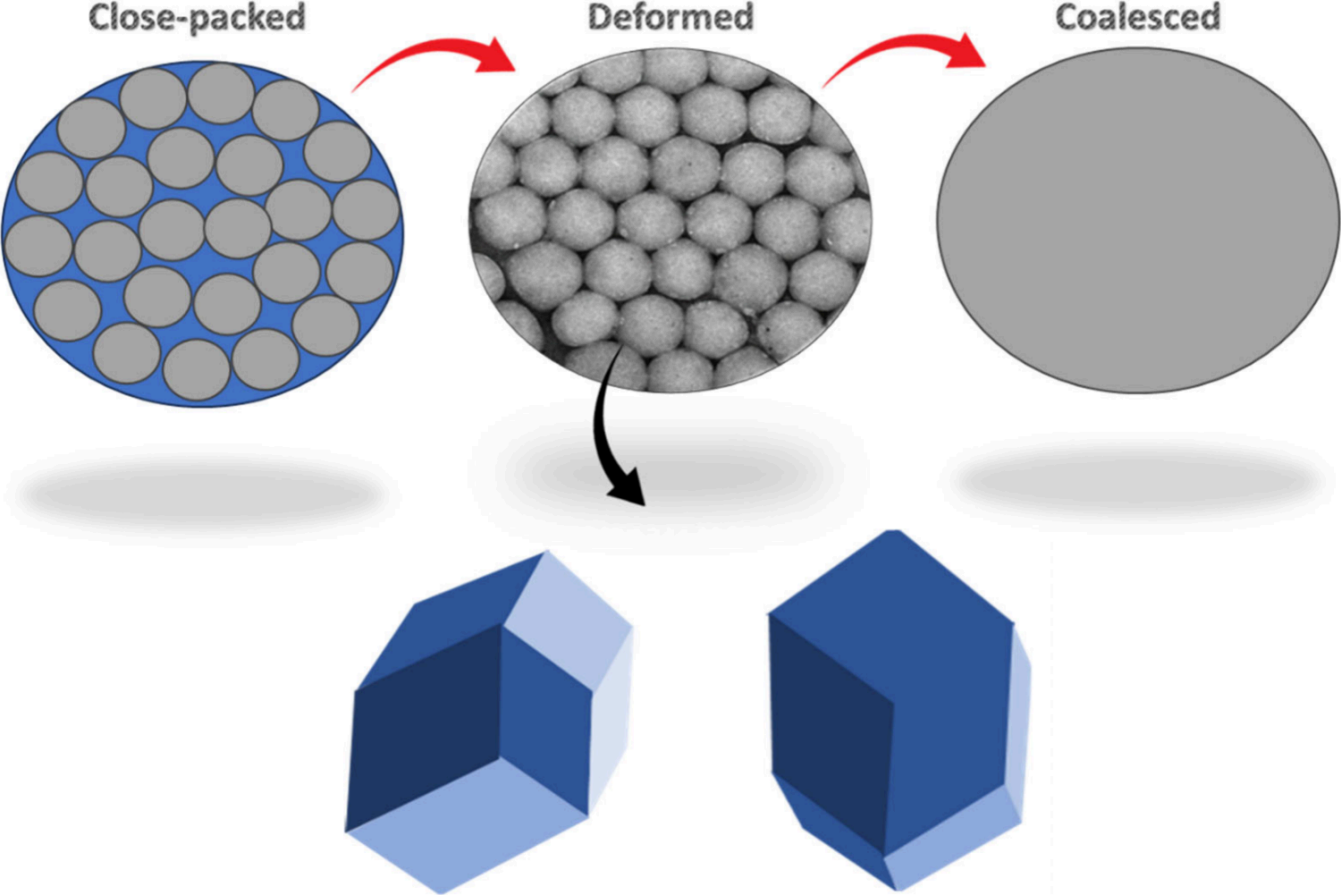
Visual evidence of the flat contact area between adjacent deformed particles, where particle identity is preserved (TEM image), and different perspectives of a rhombic dodecahedron, showcasing six square faces and six rhomboidal faces on the vertical sides, with three on the upper portion and three on the lower section. Reproduced with permission from ref (52). Copyright 2015 Royal Society of Chemistry.

Figure 4b–d showcases the cryo-SEM images of latex displaying the altered shapes of particles resulting from deformation. The once-spherical particles have now adopted nonuniform forms, with only small voids discernible at the interfaces between particles. This morphological change signifies a reduction in interparticle void space and a progression toward a denser arrangement. There are various driving forces during the film formation facilitating the deformation of particles. On the other hand, some forces are acting against particle deformation, originating from the inherent characteristics of the particles. Based on that, temperature plays a vital role in controlling the deformation phase, particularly in relation to *T*_g_.^49^ When monosized particles are oriented in the face-centered cubic array in their close-packed structure, each individual particle is in contact with 12 adjacent particles.^50^ A distinct geometric shape is formed as a result of the flattened contact areas between the particles, which is called a “rhombic dodecahedron” (illustrated in Figure 5). In this crystalline structure, each particle is surrounded by six neighbors arranged hexagonally in the (111) plane, as depicted in Figure 5. The hexagonal shape of the cross sections of the deformed particles can be evidenced by cutting the film along its (111) plane. The resulting array will resemble a honeycomb structure found in nature.

When particles possess excessive hardness, their ability to deform during film formation is impeded. This results in a cloudy and cracked film.^51^ Such a film exhibits brittleness and may even crumble into a powdery residue. To overcome this issue, the polymer must be subjected to elevated temperatures surpassing its *T*_g_. Consequently, the polymer becomes more flexible, making the film less prone to brittleness and enabling it to result in optical transparency. The minimum film formation temperature (MFFT) parameter, which is the minimum required temperature to attain optical transparency, holds significance in latex formulation. It establishes the specific conditions essential for the effective application of latex.^38^ By incorporating a blend of hard and soft particles, film formation can still occur. In this scenario, the softer particles take on the role of film formation, while the harder particles disperse throughout the film matrix.

#### 3.2.1 Particle Deformation Theories

Particle deformation theories describe the transformation soft particles undergo during close packing in processes such as the formation of latex films, driven by the goal of achieving a void-free structure while maintaining distinct particles until interdiffusion commences. This deformation process, which reduces surface free energy and surface area, is influenced by the rheological properties of the polymeric materials, temperature, drying rate, and evaporation, contributing to phenomena like colloidal crystallization. Initially, particles adopt a face-centered cubic structure, eventually transforming into a rhombic dodecahedron shape to fill space efficiently, a critical precursor to achieve mechanical strength and clarity in the material. Industrial formulators focus on optimizing this deformation step to enhance film formation and ensure the material’s suitability as a protective coating.

**Figure 6:**
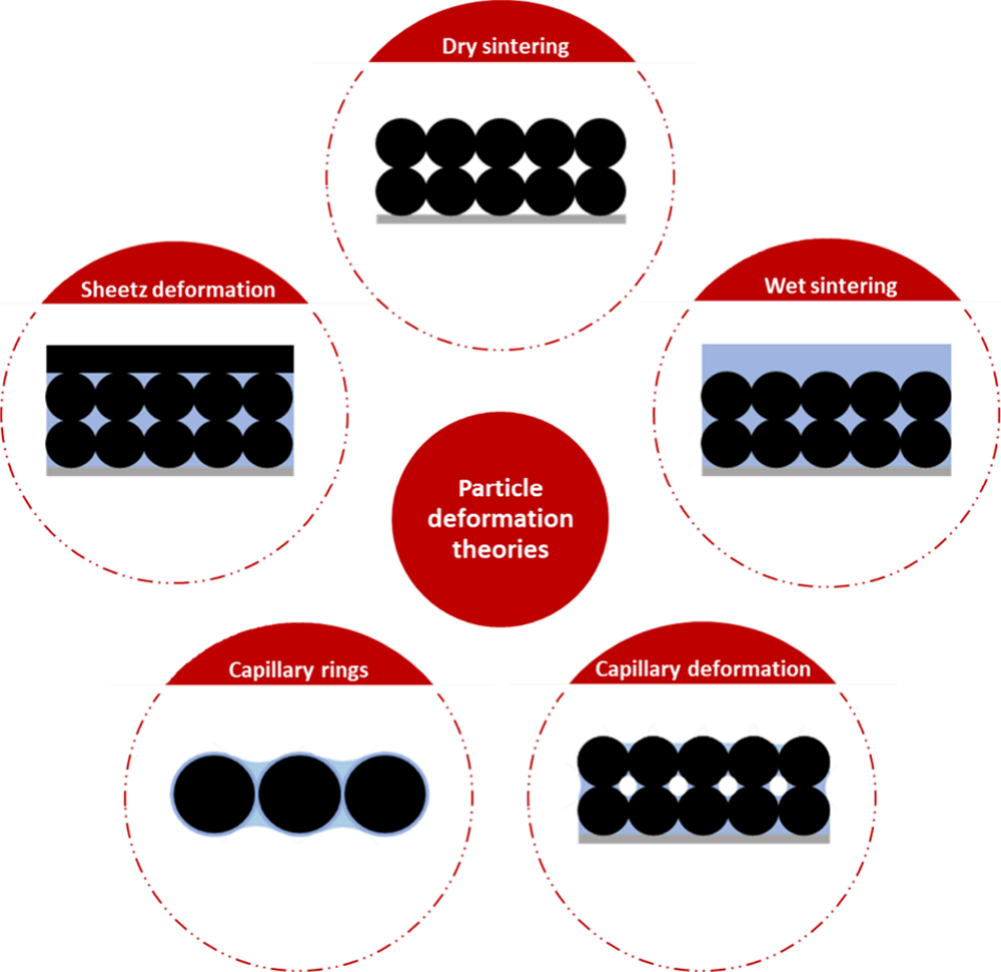
All of the proposed mechanisms contributing to particle deformation in WB coatings.

The deformation of particles during film formation is affected by various driving forces aiming to minimize interfacial area and eliminate voids between particles, a process exemplified by considering a hypothetical film scenario, as illustrated in Figure 6. This reduction in surface area, driven by mechanisms like sintering, significantly lowers the system’s overall energy. Sintering, differentiated into “dry” and “wet” types depending on the presence of water, plays a pivotal role in particle consolidation and film cohesion. Dry sintering occurs without water, facilitated by polymer–air surface tension, leading to cohesive film structures even in the absence of a liquid medium, as demonstrated in experimental studies.^53,54^ Wet sintering, on the other hand, involves particle deformation and consolidation driven by the polymer–water surface tension, with water acting as a crucial component that evaporates to bring polymer particles closer for interdiffusion.^55,56^

**Figure 7:**
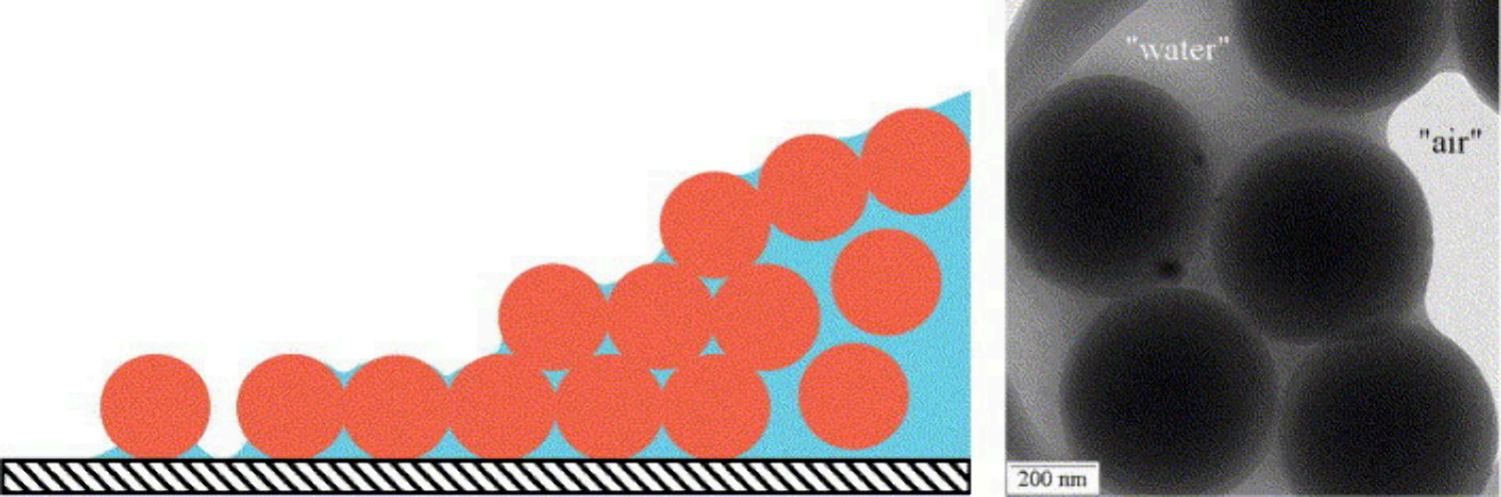
Schematically represented and experimentally observed meniscus at the surface of a wet monodisperse latex film coated on a silicon substrate. Reproduced with permission from ref (48). Copyright 2005 Elsevier.

Capillary deformation introduces another layer of complexity, driven by capillary action pulling liquid into voids between particles, shaping the film’s structure through the formation of a meniscus and resulting capillary pressure.^57,58^ The meniscus structure is depicted in Figure 7 through schematic and real representations. It is essential to note that this pressure is notably lower on the concave side of the meniscus. The capillary pressure experienced in this scenario hinges on the curvature of the meniscus. When the water’s surface forms a tight curve, it generates higher capillary pressure. Conversely, if the radius of curvature is larger, the pressure diminishes. This pressure difference that spans the meniscus can be explicitly linked to the surface tension between water and air. Notably, a higher surface tension demands a more substantial pressure to induce curvature. This mechanism is significantly influenced by the wettability of polymer particles and is a pivotal factor in the film formation process. Additionally, the presence of residual water within latex films introduces the concept of capillary rings, which encircle particle interactions, impacting the potential for dry sintering and highlighting the nuanced interplay between water and particle surfaces in film formation. Intriguingly, irrespective of the specific environmental conditions, the deformation process within a humid atmosphere featuring hydrophobic particles likely adheres to this mechanism of moist sintering. Nonetheless, the deformation process itself follows the same pattern as that observed in particles undergoing dry sintering.

**Figure 8:**
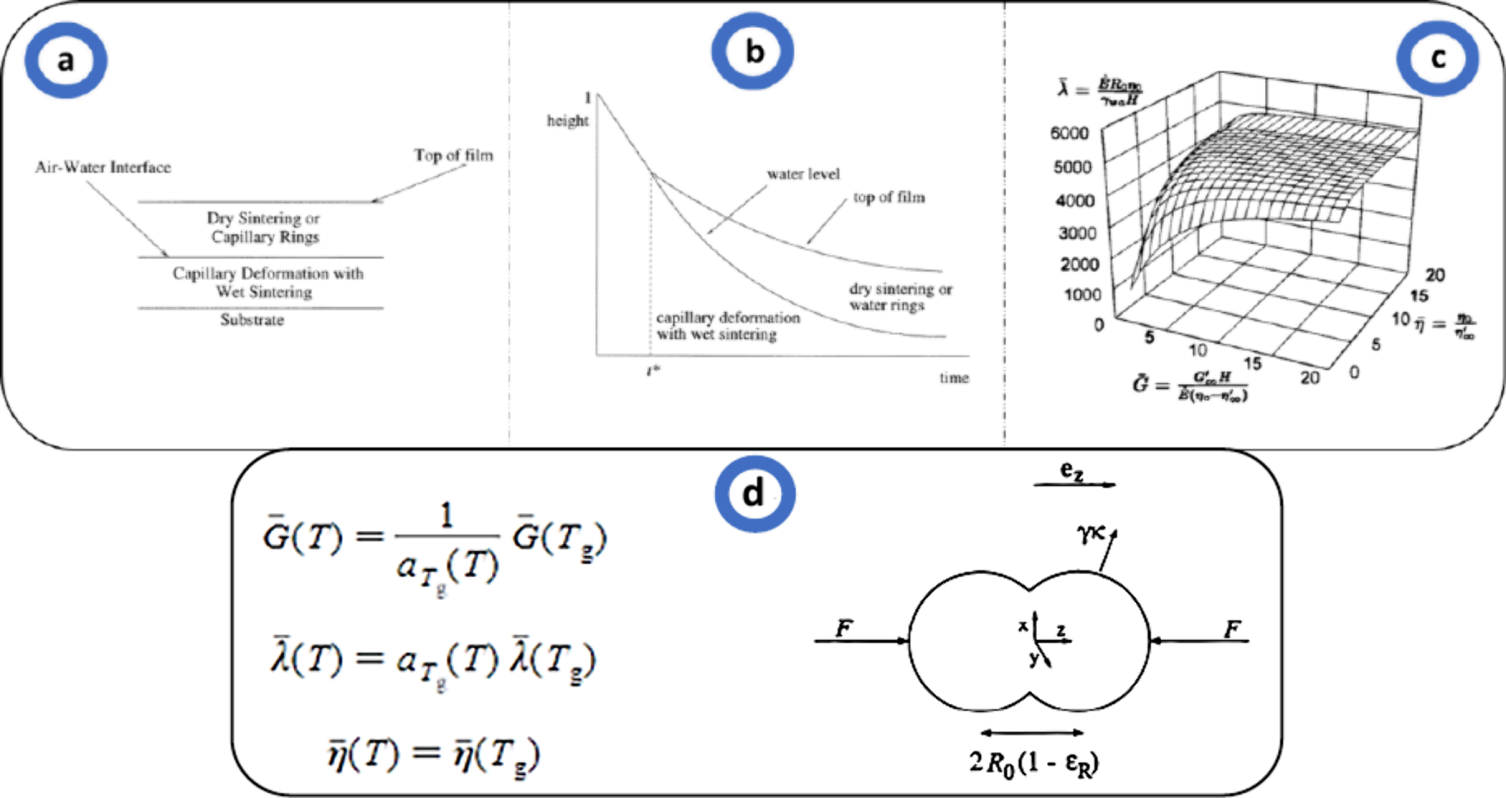
(a) Schematic of receding waterfront mechanism, (b) schematic phase diagram, (c) surface diagram of dimensionless groups, and (d) dimensionless numbers and the initial model. Reproduced with permission from ref (59). Copyright 1999 American Chemical Society.

In examining the problems with particle–particle approaches in latex film formation, the focus shifts from the interaction of isolated particles to understanding the collective behavior within the film, emphasizing the local volume fraction and the extent of particle deformation. This perspective prioritizes analyzing local stresses and the stress–strain relationship among groups of sintering particles, which is crucial for assessing how particles occupy space and contribute to the film’s structure. To achieve a comprehensive understanding, there is a need to develop a constitutive relationship that accounts for volume-averaged interactions among particles, incorporating their response to surface tension and external forces. Such a model aims to link the local volume fraction of particles to the applied stresses, offering insights into how these interactions influence the film’s architecture and properties. The deformation model proposed by Routh and Russel significantly advanced the discourse on latex film formation by integrating the viscoelastic properties of particles and accommodating multiple deformation mechanisms under varying conditions, which is illustrated in Figure 8.^45,59^ Their model, which considers the behavior of particle arrays rather than isolated pairs, elucidates the conditions under which different deformation mechanisms, such as wet and dry sintering, and capillary effects, become dominant. By identifying the maximum sustainable stress and its impact on water recession and particle deformation, the model distinguishes between scenarios of dry sintering in low humidity or high hydrophilicity and moist sintering in high humidity. This comprehensive approach, supported by differential equations and dimensionless groups, enables the prediction of dominant deformation mechanisms across different environments, enhancing the understanding of film formation processes and guiding the optimization of coating formulations.

### 3.3 Coalescence

The contact area between the particles is significantly increased during the particle deformation stage. If the ambient temperature during the film formation process is above the *T*_g_ of the polymer, the polymer chains will have sufficient mobility to undergo interdiffusion readily. The coalescence process in the film formation of WB coatings involves the movement of polymer chains across the boundaries of individual particles within the coating material as it dries or cures. This results in efficient blending of polymer chains at the particle boundaries, leading to the formation of a continuous film with sufficient mechanical properties. The diffusion process as a result of the movement of polymer chains in a constrained or entangled state is commonly referred to as “reptation”. Studies have demonstrated that the attainment of complete mechanical strength corresponds to the progress of reptation over a distance roughly equivalent to the polymer’s radius of gyration.^60,61^ While the film formation stages typically occur sequentially in localized regions, it is worth highlighting that they can concurrently take place across the entire film. Therefore, a film can exhibit fluid regions alongside fully dried and interdiffused regions, with transitional states existing between these extremes.^59^ During experimental observations, it is possible to witness drying fronts traversing laterally across films, indicating the presence of multiple states within the film. The advancement of the drying front throughout the coating, which is the dominant mechanism in the third stage of film formation, has been frequently explored in scientific literature.^62,63^ Carter et al. examined the drying front in WB coatings (free of coalescing agents) using the Routh–Russel process model under various conditions.^62^ The study reveals that at constant thickness and atmospheric conditions, lower *T*_g_ leads to skin formation, prolonging drying times. Adjusting the wet film thickness and evaporation rate influences skin formation and drying rate. Thinner films, faster initial evaporation rates, and higher polymer viscosity help avoid skin formation, improving drying efficiency.

## 4 Experimental Techniques to Explore Film Formation

Exploring processes involved in latex film formation presents a range of complex aspects that consistently captivate researchers in this domain. Every phase of film formation involves specific prerequisites, demanding the meticulous choice and application of suitable analytical methodologies. During the drying stage, precise measurement of water concentration at different points within a wet film becomes critical. However, the characteristics of latex in its wet state make techniques relying on high-vacuum conditions unsuitable, from which secondary ion mass spectrometry and Auger spectroscopy can be mentioned. Even traditional electron microscopy in its standard form falls short due to its reliance on high vacuum. Moreover, the dynamic Brownian motion of particles during drying necessitates analytical techniques that can probe without disturbing them. The ideal approach should offer insights on short time scales, while techniques capable of providing spatial profiles of particle concentration hold equal importance. During the phase where particle deformation occurs, the presence of water again imposes constraints on available techniques. The typically small diameters of particles (below 300 nm) demand high-resolution imaging methods. Understanding particle structure throughout the film, not just at the air–coating interface, becomes crucial. While scanning probe techniques and microscopies serve well for probing the air interface, obtaining information about the film’s bulk requires cross-section preparation, raising concerns about potential artifacts introduced during this process. Lastly, as the focus shifts to the interdiffusion stage, the molecular level becomes the center of attention. Noninvasive techniques capable of probing individual molecules and interfaces between particles are indispensable for unraveling the intricacies of this stage. The quest for suitable and precise analytical tools remains an ongoing endeavor for researchers in this fascinating domain.

### 4.1 Exploring the Film Formation of Wet Films

The investigation of the drying process is significantly complex due to the presence of water, especially at the early stages of a freshly applied film. To study latex films containing liquid water without disturbing the material or particle motion, various probes have been employed. These probes encompass physical probing methods (such as MFFT bar, film scratching, gravimetry, ultrasonic reflection, beam bending technique, and electrical conductivity), specialized electron microscopy techniques (such as cryo-SEM, environmental SEM (ESEM), and wet scanning transmission electron microscopy (wet STEM)), scattering techniques (such as multi-speckle diffusing wave spectroscopy (MS-DWS)), and nuclear magnetic resonance (NMR) profiling and imaging. By leveraging these diverse methods, researchers aim to unravel the intricacies of paint drying and gain deeper insights into the behavior of latex films. The principles behind them, the features, and the expected results are illustrated in Table 1 for each technique.

**Table 1:** All of the Reported Techniques with Potential/Proven Application in the Film Formation Study of WB Coatings

method	principle	features	ref
Wet Film Investigation during Film Formation			
MFFT bar	a metal bar or rod with temperature gradient is coated with a thin layer of the latex, determining the point where the coating material first becomes continuous	the MFFT bar technique is a method used to determine the lowest temperature at which a latex or emulsion coating will form a continuous film	(64), (65)
film scratching	it involves spreading a wet film on a temperature-controlled platen and using a mechanical probe to scan back and forth across the film surface	the film scratching technique provides insights into the dynamic modulus and viscosity of drying films when using a spherical probe, and it allows the observation of film hardening and cross-linking with a needle probe	(66)
gravimetry	gravimetry method involves measuring changes in the mass or weight of a sample over time as it dries or cures	this allows tracking of the solids fraction, φ, throughout the film formation process	(67)
beam bending	the film of interest is deposited onto the cantilever, and the deflection of the cantilever is measured optically; the deflection can be caused by various factors such as applied forces, residual stresses, or changes in film properties during drying or curing processes	this optical measurement provides valuable information about the mechanical behavior of the film, including its stiffness, elasticity, and response to external stimuli	(68)
ultrasonic reflection	the basic principle behind ultrasonic reflection is that the amplitude and phase of the reflected wave change depending on the properties of the film; these changes are influenced by factors such as the film’s thickness, density, porosity, and elasticity	by analyzing the reflected wave, valuable information about the film’s thickness, acoustic impedance, and other related properties can be obtained	(69)
electrical conductivity	the method involves inserting metallic wires into the wet film or attaching electrodes to a substrate and spreading the film across them; by measuring the electrical conductivity of the film, valuable information about its properties can be obtained	the conductivity of the foam is related to its water content, providing insights into the foam structure; conductivity measurements can also indicate the presence of particle boundaries in a dried film that has reached optical clarity	(70)
cryo-SEM	by maintaining the sample at extremely low temperatures, typically below −150 °C, water is transformed into an amorphous solid state (the sample preserves its native structure)	by freezing the sample, it preserves its natural state, providing high-resolution imaging with minimal distortion; it allows for the visualization of delicate structures	(71)
	the frozen sample is then transferred to the cryo-stage of the SEM		
ESEM	in ESEM, the sample chamber is equipped with a specialized environmental cell that allows the introduction of a controlled gas environment; the chamber is designed to maintain a specific relative humidity and gas composition throughout the imaging process	the key advantage of ESEM is its ability to image samples under near-natural or in situ conditions	(72)
wet STEM	in wet STEM, the sample is prepared by placing a liquid droplet or thin film onto a specialized liquid cell; the liquid cell is typically composed of thin, electron-transparent windows, such as silicon nitride or graphene, which allow the transmission of electrons while maintaining the liquid sample	it allows researchers to visualize the dynamic behavior of wet or hydrated samples in their native liquid environment	(73)
MS-DWS	in MS-DWS, a laser beam is directed at the wet film, and the scattered light is collected and analyzed; the scattered light forms speckle patterns due to the interference of light waves scattered by different particles within the film	by analyzing the temporal fluctuations in the speckle pattern, information about the motion and dynamics of the particles in the film (mechanisms and kinetics of film formation) can be extracted	(74)
NMR	NMR profiling involves the application of a strong magnetic field to a wet film sample, causing the nuclei of certain atoms within the sample to align with the field; by varying the magnetic field and employing gradient coils, NMR imaging generates a two-dimensional or three-dimensional representation of the film’s internal structure	NMR profiling and imaging provide nondestructive, noninvasive, and quantitative information about the drying process and the resulting film morphology	(75)
Particle Packing and Deformation in Dry Films			
AFM	it operates by scanning a sharp tip (probe) over the surface of a sample while measuring the interaction forces between the tip and the sample	it enables the visualization of surface topography with subnanometer resolution, measurement of surface roughness, mapping of mechanical properties such as stiffness and adhesion, and even manipulation of individual atoms and molecules	(76)
SNOM	in SNOM, a light source is focused onto the back of the probe tip, and as the tip approaches the sample, a small aperture at the tip’s apex confines the light to a subwavelength spot; the interaction between the sample and the near-field light generates a signal, such as light scattering, fluorescence, or absorption, which is detected by the tip and used to generate an image	SNOM enables the investigation of optical properties, such as local refractive index variations, fluorescence emission, and light–matter interactions, at the nanoscale	(77)
TEM	a beam of electrons is generated and accelerated through an electron gun; the electrons pass through electromagnetic lenses that focus the beam onto the sample; as the electrons interact with the sample, they undergo different interactions, including scattering, diffraction, and absorption, which are collected then using a detector	it can provide atomic-level details of crystal structures, lattice defects, grain boundaries, and interfaces	(78), (79)
SEM	in SEM, a beam of electrons is generated and accelerated through an electron gun; the electrons pass through electromagnetic lenses that focus the beam onto the sample surface; as the beam interacts with the sample, various signals are emitted or generated, which are collected then using a detector	SEM provides high-resolution imaging, allowing for detailed examination of surface features, such as cracks, pores, particles, and textures; it offers a large depth of field, enabling the imaging of samples with uneven surfaces	(80)
Interdiffusion and Coalescence			
SANS	it involves the interaction of neutrons with the sample’s atomic and molecular structures; the intensity and scattering angles of neutrons are sensitive to the size and spacing of nanoscale structures in the sample	the scattering pattern reveals valuable details about the interparticle distances, interconnectivity, and clustering behavior; this information helps in understanding the interdiffusion kinetics and the formation of hierarchical structures within the film	(81)
FRET	the working principle of FRET is based on the transfer of energy between two fluorophores (molecules that emit light when excited by a specific wavelength) when they are in close proximity, typically withing a range of 1–10 nm, and in a suitable orientation	by monitoring the FRET signal between the donor and acceptor dyes, it is possible to observe the extent and kinetics of interdiffusion and coalescence; as interdiffusion and coalescence occur, the FRET efficiency may change, indicating the degree of mixing and blending within the film	(82)

### 4.2 Exploring the Film Formation of Dry Films

Numerous techniques have proven effective in providing insights into the distribution of particles within wet films. These techniques can be extended to characterizing the particle arrangement and behavior as the film undergoes the initial stages of packing and deformation. However, there are alternative techniques that, due to specific constraints, cannot be employed to study wet films. These techniques can be classified as electron microscopies (such as TEM and SEM), scanning probe microscopies (such as atomic force microscopy (AFM), scanning electric potential microscopy (SEPM), and electric force microscopy (EFM)), shear force microscopy, and scanning near-field optical microscopy (SNOM). More information related to these techniques is illustrated in Table 1.

### 4.3 Exploring the Interdiffusion and Coalescence

**Figure 9:**
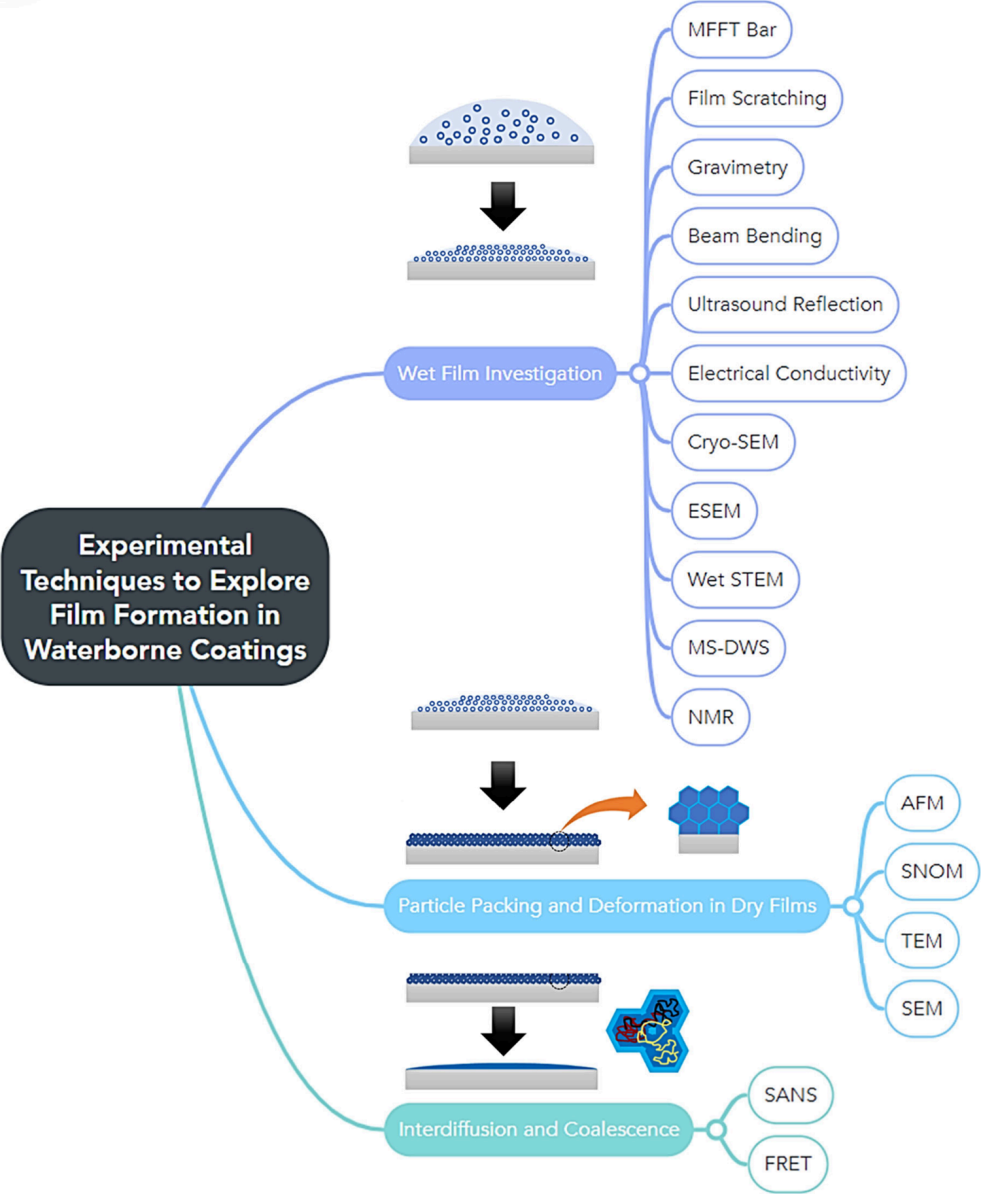
A mind map of different experimental techniques to explore film formation in WB coatings categorized by each stage.

Interdiffusion takes place as the water evaporates, leading to the diffusion of polymer chains across their boundaries during the last phase of film formation. Interdiffusion is a mandatory step for the development of the structure, morphology, and mechanical properties of the final film. Studying interdiffusion and coalescence in WB coatings requires a combination of specialized techniques that can provide insights into the molecular and morphological changes occurring during the film formation process. Small-angle neutron scattering (SANS) proves to be a valuable method for examining the interdiffusion and coalescence processes that take place during the development of WB films. SANS allows researchers to study the kinetics and extent of interdiffusion by analyzing the scattering patterns of neutrons. Fluorescence resonance energy transfer (FRET) stands out as a potent method employed for the investigation of molecular interactions, conformational changes, and distances between biomolecules or fluorophores. The principles behind them, the features, and the expected results are illustrated in Table 1 for each technique, and the corresponding mind map is illustrated in Figure 9.

### 4.4 Experimental Evidence

**Figure 10:**
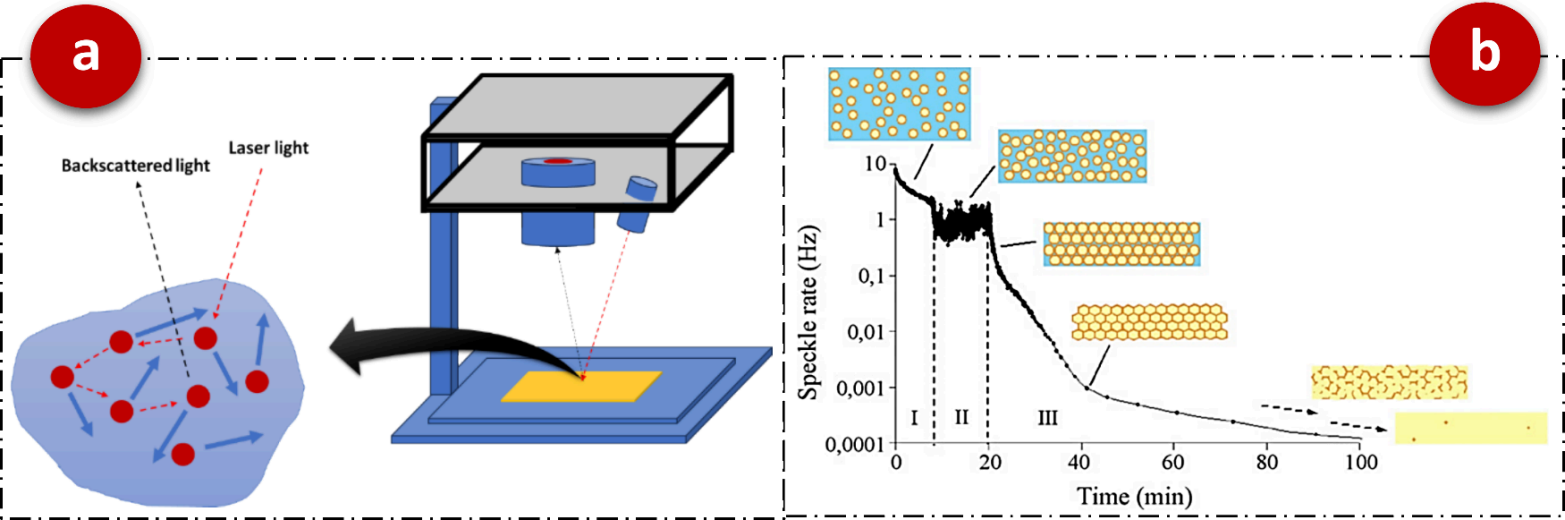
(a) Schematic representation of the MS-DWS setup to study the film formation through a nondestructive, in situ procedure. (b) Kinetics of A.S.I.I. observed during the formation of a WB sample on a glass plate, illustrating distinct phases. Reproduced with permission from ref (74). Copyright 2008 Elsevier.

While theories are important and act as the basis for any research, it is essential to visualize the mechanisms governing film formation in practice. To give a practical aspect to the knowledge formed in theories, there are some worth reading, specific reports in literature using some of the techniques summarized in the last section to study the film formation of WB coatings. Brun et al. introduced the use of MS-DWS for studying the film formation process of WB coatings, marking a significant advancement in coating research.^74^ Leveraging the principles of dynamic light scattering, MS-DWS has been instrumental in analyzing the microrheology of colloidal gels and the gelation phenomena in various food products.^83−85^ By applying this technique to the drying and curing phases of coating formulations, the team aimed to correlate the kinetics of film formation, as observed through MS-DWS, with conventional analytical methods such as gravimetric analysis and manual testing. This innovative approach allowed for real-time monitoring of structural changes within the coating, capturing the dynamics of particle motion through the analysis of backscattered light and speckle rate changes, which decrease as the coating transitions through concentration, packing, and consolidation stages due to solvent evaporation and particle interaction (Figure 10a).^86,87^

The film formation process of coatings can be systematically analyzed through Adaptive Speckle Imaging Interferometry (A.S.I.I.) processing of backscattered light, revealing a three-phase kinetic model: concentration, packing, and consolidation stages (Figure 10b). This is a patented processing technique developed by the same group, embedded since 2006 in a commercially available instrument (schematically shown in Figure 10). Initially, high speckle rates indicate rapid particle movement within the film due to solvent evaporation, leading to the Brownian motion of scatterers. As drying progresses, fluctuations in speckle rate mark the transition to the packing stage, where the decrease in water content and increase in particle concentration cause particles to impede each other’s movement, reminiscent of jammed colloidal behavior. The final consolidation phase is characterized by a significant drop in speckle rate, reflecting decreased particle motion as the film becomes more coherent and water is fully evaporated. This model underscores the nonlinear nature of particle dynamics during drying, highlighting the intricate balance of evaporation, particle interaction, and coalescence in achieving a cohesive film.

The research further explored the influence of substrate properties on the film formation process by applying consistent paint layers on different substrates, including gypsum drywall, glass, and medium-density plywood. The findings highlighted the impact of substrate porosity on the drying kinetics, where porous materials like plywood and gypsum exhibited a faster decrease in speckle rate compared to glass, indicating quicker immobilization of scatterers within these substrates. This absorption effect, combined with the substrate’s scattering properties, affected the overall drying time and the film’s structural development. The study underscores the complexity of film formation, influenced by substrate characteristics, and demonstrates how MS-DWS can provide valuable insights into the drying behavior and kinetics of WB coatings on various surfaces.

**Figure 11:**
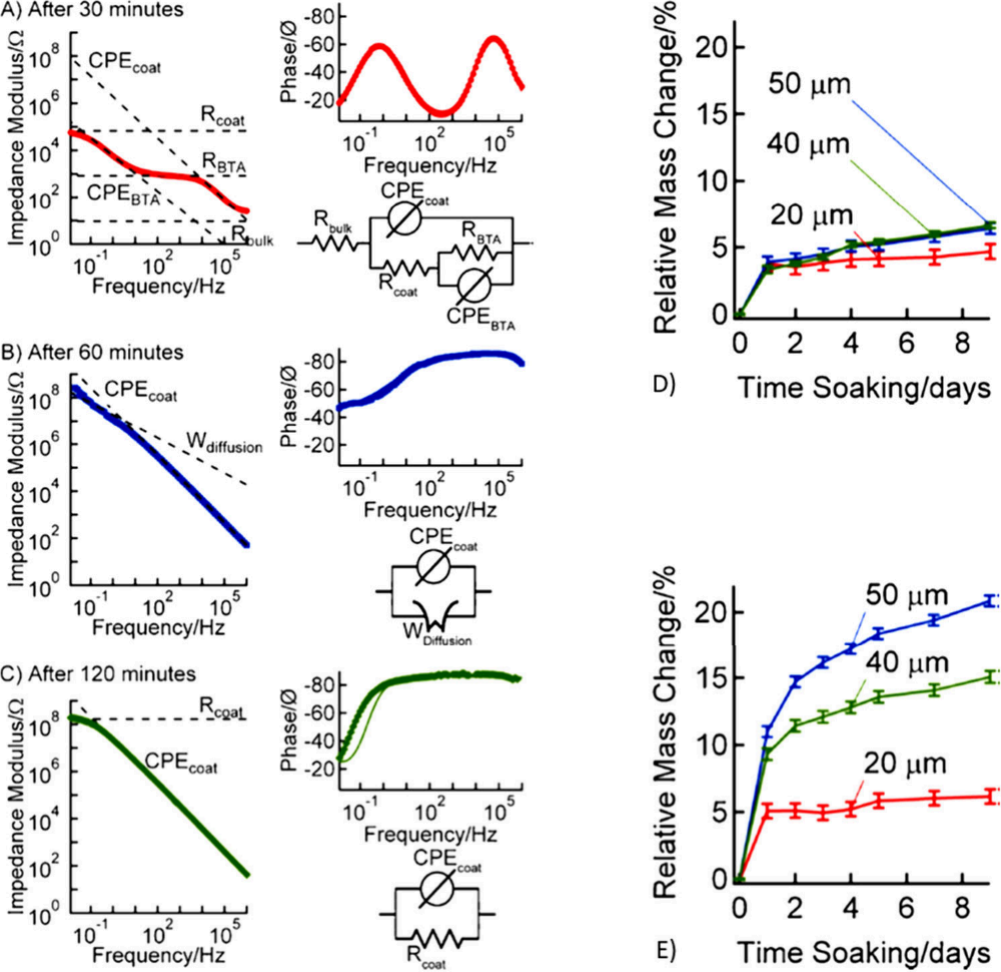
Bode plots of WB film formation with data fitted to models featuring the following circuit elements: (a) tacky film (30 min): *R*_coat_ = (6.88 × 10^4^) ± (1.29 × 10^3^) Ω, CPE_coat_ = (106.4 × 10^–9^) ± (7.14 × 10^–9^) S s^α^, α = 0.874 ± 0.00529; (b) semidry film (60 min): CPE_coat_ = (5.87 × 10^–9^) ± (7.24 × 10^–11^) S s^α^, α = 0.874 ± 0.00529, *W*_diffusion_ = (21.1 × 10^–9^) ± (1.70 × 10^–10^); (c) completely dry film (120 min): *R*_coat_ = (1.69 × 10^8^) ± (1.49 × 10^6^) Ω, CPE_coat_ = (7.55 × 10^–9^) ± (4.74 × 10^–11^) S s^α^, α = 0.948 ± 0.00670, and water absorption profile of (d) SB and (e) WB films with varying thickness during a 9-day immersion period. Reproduced with permission from ref (19). Copyright 2012 Elsevier.

Electrochemical impedance spectroscopy (EIS) stands as a venerable technique in the realm of studying the anticorrosion efficacy of organic coatings. With a rich body of literature supporting its utility,^88−92^ EIS emerges as a pivotal tool offering direct and sensitive insights into corrosion phenomena occurring on coated metal substrates. Beyond merely detecting corrosion reactions, EIS unveils a trove of information concerning the protective coating’s intrinsic properties. These include, but are not limited to, dielectric properties, porosity of the film, and the extent of ion diffusion.^93,94^ Over the past few decades, EIS instrumentation has undergone significant advancements, reaching a pinnacle where it now affords reliable measurements even for high-impedance protective coatings. This technological evolution marks a pivotal juncture, expanding the utility of EIS beyond its traditional role in evaluating anticorrosion coatings. Now, EIS finds itself at the forefront of a broader spectrum of protective coatings research and development endeavors. Its newfound capability to ascertain film properties in the absence of discernible substrate corrosion extends its applicability, underscoring its relevance in modern materials science and engineering. Swartz et al. investigated the two comparable WB and SB coatings applied on bronze substrates using EIS, which is a nonconventional method for such a purpose, to understand their film formation mechanisms.^19^ The focus was on physical processes rather than chemical composition. WB coatings were formulated using a commercial acrylic copolymer containing methacrylic acid, methyl methacrylate, and butyl acrylate. SB coatings were formulated using a commercial binary copolymer resin, mainly composed of methacrylate and ethyl acrylate in toluene. To monitor their impedance during the drying process, they were applied onto bronze substrates. For the WB coating, a flash-rust inhibiting layer (BTA) was applied on the substrate before the WB coating. Based on the results illustrated in Figure 11, the SB coatings dried rapidly, with a tacky stage within 5 min and a dry stage at 15 min, with different impedance characteristics observed. It was shown that the film formation of these two types of coatings follows different mechanisms.

WB coatings differ from SB coatings by requiring an additional coalescence step for film formation. Through impedance spectroscopy over two hours, changes in the coatings’ properties were monitored, revealing that the bulk solution resistance (∼9 Ω) is negligible in systems with SB coatings. The initial resistance measurements showed the coating and the BTA layer with resistances of 68.8 and 0.836 kΩ, respectively. Further analysis at the 60 min mark, when the WB film appeared dry to touch, indicated ongoing diffusion processes, highlighting that barrier properties at this stage are less effective compared to those observed after 120 min of drying. The final film exhibited significantly improved barrier properties, with a resistance of 170 MΩ, closely aligning with fully dried SB coatings. This suggests that the barrier properties of WB coatings can improve over time, initially increasing after 30 days due to the slow diffusion of trapped additives, although prolonged aging may reduce these properties due to polymer degradation. Moreover, EIS analysis underlined the enhancement in barrier properties against electrolyte penetration after 120 min of air drying, with films demonstrating substantial resistance to electrolyte uptake. A study on the correlation between barrier properties and film thickness showed that WB films absorb more water than SB films, with thicker WB films exhibiting higher water absorption rates. Despite prolonged immersion, the thickest WB films did not reach saturation, contrasting with SB films, which showed minimal water absorption variance with thickness. These findings underscore the dynamic nature of film formation and drying in WB coatings, indicating that their barrier properties evolve with drying time and are influenced by film thickness, offering valuable insights for optimizing coating formulations for improved durability and performance.

Both WB and SB coatings undergo two distinct phases of water sorption: a rapid initial uptake where at least 50% of the total water mass is absorbed within the first 24 h, followed by a more gradual absorption process until saturation is reached. WB coatings exhibit a higher rate of water uptake compared to SB coatings, attributed to their hydrophilic nature. After the first day, the water absorption rate decreases, although thicker films, achieved through multiple applications, tend to absorb more water overall due to their heterogeneous pore and void structures. EIS and gravimetric analysis reveal that WB films have higher water and electrolyte uptake than do SB films. Discrepancies between EIS data and gravimetric results suggest complex water penetration mechanisms, involving both surface and bulk absorption. Upon annealing, both WB and SB films demonstrate improved barrier properties, with WB films showing more significant enhancement. This improvement is likely due to the healing of defects, the evaporation of hydrophilic components, and the completion of coalescence processes. The researchers noted that the presence of a top layer in latex films might slow the diffusion of coalescing agents, an observation supported by the continued improvement in resistance observed in films even after 30 days of drying. Annealing is proposed to further benefit WB film formation by facilitating coalescence and the removal of hydrophilic components, underscoring the importance of postapplication treatment in optimizing the performance of coating films.

Using the same methodology, EIS results accompanied by AFM images were utilized by Berce et al. to monitor the film formation of commercial WB latex coatings.^95^ Considering the impedance and Warburg element of the coating for 2 weeks, it was possible to determine how much the film formation and close-packing/coalescence progressed at different time points. The conclusions made by EIS results on the system were also visually verified using AFM images. One issue with this method applied for such a purpose is the need for a relatively dry film to start the measurement. Therefore, the EIS technique can be used as a tool to study the film formation after the bulk water evaporation from the freshly applied film, which mostly covers stages II and III.

## 5 Coalescence Challenges and Solutions

Considering all of the mechanisms proposed for the film formation of WB coatings, there is a paradox that arises from the conflicting requirements in certain applications of latex films. For instance, the film formation of household paints needs to happen at room temperature, requiring the polymer *T*_g_ value to be lower than ambient conditions. However, these applications also demand durable and scratch-resistant surfaces. The incorporation of hard particles is one common solution to achieve this property. Paradoxically, to effectively bind these hard particles together, a soft polymer binder is still needed. To overcome this challenging and at the same time essential stage in the film formation of WB coatings, there are some conventional and futuristic (environmentally friendly) solutions discussed in this section.

### 5.1 Key Role of Coalescing Agents

**Figure 12:**
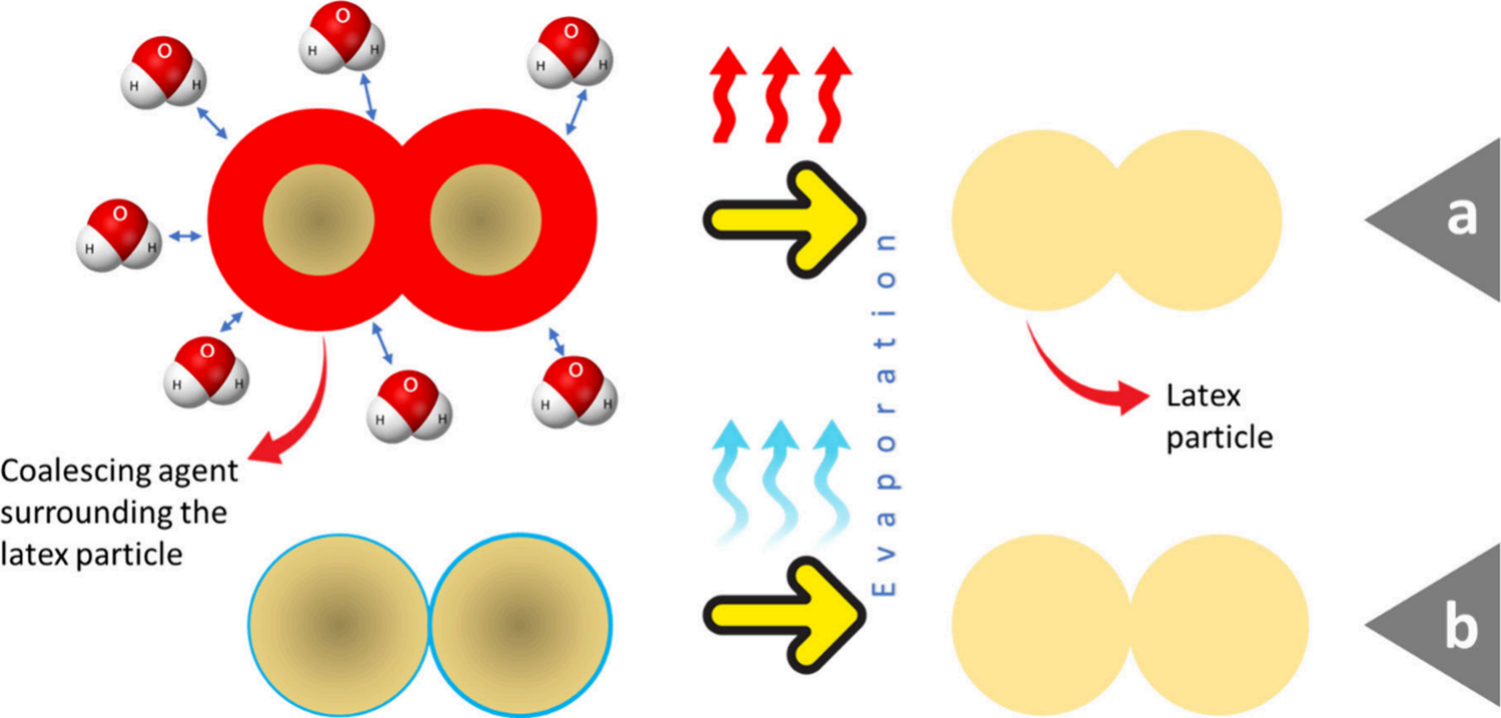
A schematic representation of the mechanism through which coalescing agents facilitate the coalescence of latex particles: (a) how coalescing agents leave behind the latex particles after evaporation and (b) the corresponding phenomenon without coalescing agents.

It is impossible to create films solely from latex particles with a *T*_g_ above ambient temperature. To resolve this paradox, one common approach involves lowering the *T*_g_ by introducing diluent molecules, often referred to as “plasticizers”, “coalescing aids”, or “coalescing agents”. Typically, these compounds are composed of volatile molecules to facilitate film formation and subsequently evaporate, leaving behind a film with a higher *T*_g_, which is schematically depicted in Figure 12. One frequently utilized coalescing agent found in the literature is 2,2,4-trimethyl-1,3-pentanediol monoisobutyrate (TPM), commercially recognized as TexanolTM.^96^ The influence of a coalescing agent encompasses more than just the reduction of the polymer latex’s *T*_g_. Diffusivity experiences a significant boost as the temperature surpasses the *T*_g_ threshold. It is precisely this heightened diffusivity that empowers the formation of continuous and robust films. When choosing a coalescing agent, several factors should be taken into account, as outlined below:

(1) Evaporation rate: The time required to reach the maximum film hardness is directly affected by the evaporation rate of the coalescing agent. Even small amounts of the coalescing agent are potentially enough to soften the coating. This phenomenon becomes evident in FRET experiments, where it was observed that as the evaporation of the coalescing agent proceeds, the diffusivity of poly(butyl methacrylate) latex particles decreases.^97^
(2) *T*_g_: Selecting a coalescing agent with the appropriate *T*_g_ reduction is essential. If the *T*_g_ is lowered too much, the coating may remain tacky or have poor mechanical properties. If the *T*_g_ reduction is insufficient, film formation may be incomplete. The *T*_g_ can be estimated to be approximately two-thirds of the absolute melting temperature (in kelvin).^98^ For instance, the incorporation of 10 wt % TPM can lower the *T*_g_ of poly(butyl methacrylate) to as low as 10 °C, while using the same concentration of diacetone alcohol will lower it to 26 °C.^99^
(3) Solubility: Coalescing agents tend to partition between the polymer phase and the aqueous phase in the wet latex. The plasticizing effect of a coalescing agent is delayed until it moves to the polymer phase. Therefore, the higher is the aqueous solubility of coalescing agents, the lower is their efficiency. The degree of partitioning plays a crucial role in determining the number of solvent molecules in both phases, thus impacting the level of plasticization. For example, TPM has low solubility in water and mainly resides in the polymer phase. As a result, it can enhance diffusivity when particles initially come into contact, even in the presence of water.

**Figure 13:**
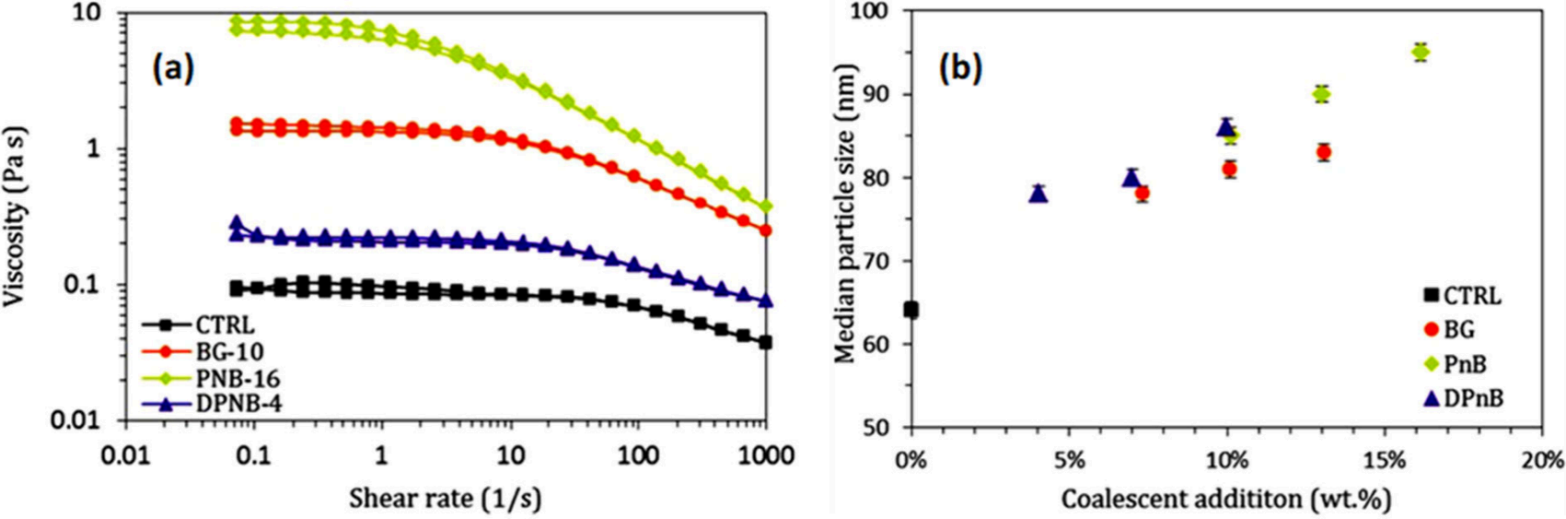
(a) Flow curves of various dispersions and (b) median particle size versus the loading concentration of the coalescent. Reproduced with permission from ref (15). Copyright 2017 John Wiley and Sons.

Berce and colleagues conducted a comprehensive study on the effects of hydrophilic and hydrophobic coalescing agents on WB coating formations, utilizing an extended approach based on EIS.^15^ Their experiments, which varied the amount of coalescing agents added to a latex dispersion, aimed to assess the impact on film formation dynamics and quality, using an extended approach based on a recently developed EIS technique.^95^ It was found that the control sample without coalescing agents failed to form a homogeneous film under room temperature conditions. Different curing conditions were tested to explore their effects on the film formation process, revealing that the drying and curing environment significantly influenced the film’s development. The addition of coalescing agents notably altered the physical properties of the coatings, including viscosity changes as depicted in Figure 13a, with hydrophilic and hydrophobic agents impacting the system differently. Notably, the hydrophobic agents PnB (1-butoxy-2-propanol) and DPnB (di(propylene glycol) butyl ether) increased particle size more significantly than the hydrophilic BG (2-butoxyethanol) agent as indicated in Figure 13b. It was suggested that the viscosity was increased due to the coalescent partitioning inside the polymer particles, which resulted in an enhanced volume fraction of the polymer phase.^100^

The study further delved into the electrical properties and structural changes in the films, finding that all coalescing agents led to reductions in pH, conductivity, zeta potential, and surface tension, with hydrophilic agents having a more pronounced effect. This was attributed to the formation of a solvation layer around the particles, affecting the emulsifier’s conformation and leading to reductions in various measured properties. Moreover, the drying profiles indicated a uniform “set-to-touch” time across samples, with higher concentrations of coalescing agents generally resulting in more rigid films. However, excessive amounts of certain agents negatively affected film hardness, highlighting the importance of optimizing coalescing agent concentrations for desired film properties.

EIS analysis provided insights into the mechanical properties and ion diffusion within the films, with distinct behaviors observed for different coalescing agents. The optimization study aimed at improving the cost-effectiveness of WB coatings for metal substrates indicated that formulations combining BG and DPnB offered the best rapid-drying capabilities. However, longer drying times at moderate temperatures were found to be more indicative of expected performance, underscoring the need for careful consideration of drying conditions in evaluating WB coating performance. This research highlights the complex interplay between coalescing agents, drying conditions, and the resultant physical and mechanical properties of WB coatings, offering valuable insights for the development of optimized coating formulations.

### 5.2 Futuristic Coalescing Agents

Despite the shift to WB coatings, which use water as a solvent to reduce VOC emissions, the incorporation of coalescing agents, which aid in the film formation process, remains a challenge. These agents, necessary for achieving optimal coating properties such as gloss and scrub resistance, eventually evaporate, releasing VOCs into the environment. This ongoing emission from coatings is problematic, particularly given the vast global market for WB coatings, which continues to grow. The need for safer, more sustainable alternatives to traditional VOC-based coalescing agents is driven by increasing regulatory pressures, environmental concerns, and the demand for more eco-friendly coating formulations.

Kaur and co-workers introduced a novel approach to improving film formation in WB coatings without relying on traditional VOC-based coalescing agents.^101^ They developed a series of reactive coalescing agents based on hydroxyethyl sulfone compounds, which undergo dynamic equilibrium with vinyl sulfone. These hydroxyethyl sulfone-based reactive coalescing agents were synthesized by connecting hydroxyethyl sulfone moieties to conventional coalescing agents and were characterized through various analytical methods. The key innovation lies in the ability of these reactive coalescing agents to remain inert during storage and application but react with latex polymers during the film formation process without requiring external triggers like heat or UV light. This reactivity is driven by the formation of vinyl sulfone from hydroxyethyl sulfone under basic conditions, which then undergoes Michael addition with nucleophiles present in the latex. The study demonstrated that these reactive coalescing agents effectively lower the MFFT of commercial lattices, facilitating film formation at lower temperatures. The vinyl sulfone intermediates were isolated and characterized, confirming their role in the cross-linking process. Moreover, the hydroxyethyl sulfone-based reactive coalescing agents showed significantly reduced weight loss compared to their nonreactive analogs, indicating lower VOC emissions. The reactivity studies further confirmed that these reactive coalescing agents integrate into the polymer matrix during drying, leading to stable, environmentally benign coatings. This innovative approach offers a promising alternative to traditional VOC-based coalescing agents, potentially revolutionizing the formulation of waterborne coatings by enhancing performance while reducing environmental impact.

A green method was developed by Dogan-Guner et al., to enhance the mechanical performance of WB acrylic coatings without adding coalescing agents.^102^ By incorporating unmodified cellulose nanocrystals into the acrylic latex, they avoided the need for surface functionalization or copolymerization. By incorporating cellulose nanocrystals, which have strong hydrogen-bonding potential due to their hydroxyl groups, the researchers were able to enhance interactions between cellulose nanocrystals and the carboxylic acid groups present in the latex particles (from methacrylic acid). This interaction promoted better dispersion of cellulose nanocrystals within the latex matrix, preventing their aggregation and ensuring a more homogeneous distribution throughout the film. As the latex particles coalesced during drying, cellulose nanocrystals became confined in the interstitial spaces between particles, leading to a network that increased the mechanical strength of the films without needing coalescing agents. The cellulose nanocrystals also improved the film’s tensile strength, modulus, and hardness by forming a reinforcing network within the matrix, which provided enhanced mechanical properties typically associated with harder, VOC-containing coatings.

In another environmentally friendly attempt, Dron et al. reduced the amount of VOC-based coalescing agents in high-performance WB coatings through the in situ creation of oligomers using 2-ethylhexyl thioglycolate as a chain transfer agent during polymerization.^103^ The innovative approach involved introducing this chain transfer agent toward the end of the polymerization process, which led to the formation of temporary oligomer plasticizers directly within the latex particles. The key improvement mechanism revolves around these oligomers’ ability to lower the MFFT of the latex films. The oligomers, created by the chain transfer agent, act as temporary plasticizers by disrupting the polymer’s intermolecular forces, thereby facilitating easier fusion of polymer chains during film formation. This results in a significant reduction in the MFFT, as evidenced by the substantial decrease observed in the latices containing the oligomers compared to standard formulations. The mechanism behind this improvement involves the oligomers modifying the polymer matrix’s local mobility, allowing the latex particles to coalesce more effectively at lower temperatures. The study demonstrated that this method not only reduced the MFFT but also preserved the final film’s mechanical and water resistance properties, as confirmed by various characterization techniques. This approach offers a sustainable alternative to traditional coalescing agents by achieving comparable film formation enhancements through the strategic use of in situ generated oligomers.

Following the review of various strategies for improving film formation in WB coatings, several methods show promise in addressing the challenges of VOC-based coalescing agents. Hydroplasticization, leveraging water’s role in film formation, offers benefits but is hindered by variability in ambient drying conditions.^104^ Cross-linking low *T*_g_ polymer lattices can produce durable films, yet it requires precise timing to avoid issues with gel content.^105^ Supramolecular chemistry introduces noncovalent bonding techniques, such as hydrogen bonding and electrostatic interactions, although these may suffer from weak bonds or interference from other components like surfactants.^106−108^ A particularly promising alternative involves using oligomers as temporary plasticizers, as discussed earlier.^103^ These oligomers can effectively mimic the role of traditional plasticizers without evaporating, but their successful implementation depends on their incorporation into latex particles. Achieving this requires the use of chain transfer agents with a balanced hydrophobicity to facilitate in situ oligomer synthesis during polymerization. For all of these methods to fully replace VOC-based coalescing agents in commercial products, it is essential to not only enhance film formation efficiency and lower MFFT but also to ensure that the mechanical properties of the final film remain intact. Continued research and optimization in these areas are crucial for advancing these eco-friendly alternatives into widespread industrial use.

## 6 Different Environmental Parameters Controlling the Coalescing Stage

The coalescing stage in WB coatings film formation is crucial, as it determines the final film’s properties, such as its appearance, durability, and resistance to environmental factors. This stage is influenced by various environmental parameters, each playing a vital role in ensuring the successful transition from dispersed particles to a coherent film. Understanding these parameters can help optimize the film formation process and improve coating performance. Temperature significantly affects the rate of water evaporation and the mobility of polymer particles. Higher temperatures can accelerate the evaporation of water and the coalescing agent, leading to faster film formation. However, too high a temperature might cause rapid surface drying, trapping water, and coalescing agents within the film, potentially leading to defects. Ambient humidity influences the evaporation rate of water from the coating. High humidity can slow the evaporation process, extending the time required for film coalescence and potentially affecting film integrity. Conversely, low humidity conditions can lead to quicker water loss, which might not provide sufficient time for particles to coalesce properly. The flow of air over the coating surface can enhance the evaporation of water and coalescing agents, influencing the drying and coalescing rates. Proper ventilation helps remove the evaporated water from the coating environment, but excessive airflow might cause uneven drying.

Chen et al. conducted a detailed study on the impact of temperature and relative humidity on the film formation process of polymeric latex dispersions, revealing a three-stage process: Stage I*, Stage I**, and Stage II*.^109^ The initial stage, Stage I*, involves the beginning of latex particle crystallization, leading to a face-centered cubic structure in Stage I** as particles interact more closely. The final stage, Stage II*, is characterized by the partial deformation and compaction of particles at temperatures above *T*_g_, resulting in incomplete water evaporation. Utilizing a styrene/*n*-butyl acrylate copolymer (PS-*co*-BA) stabilized with negative surface charges and polyelectrolytes, the study employed advanced techniques like SAXS for sizing and distribution analysis, and TGA for monitoring weight changes under different environmental conditions, demonstrating that drying temperature and humidity significantly influence the evaporation rate and subsequent film properties.

**Figure 14:**
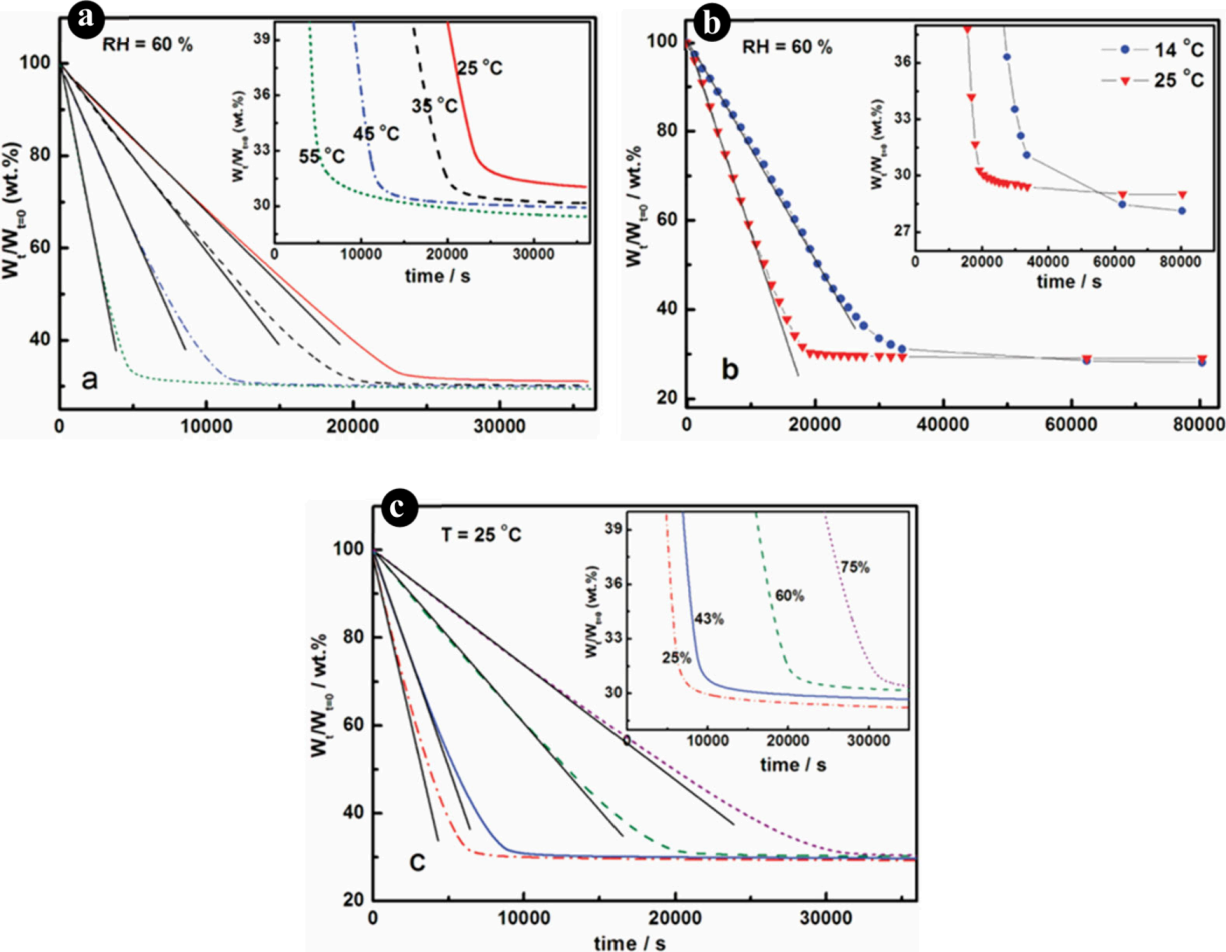
Evaporation rate based on the relative weight of the sample compared to the initial state resulted from the TGA analysis: (a,b) various temperatures (constant relative humidity) and (c) relative humidities (constant temperature). Reproduced with permission from ref (109). Copyright 2011 American Chemical Society.

**Figure 15:**
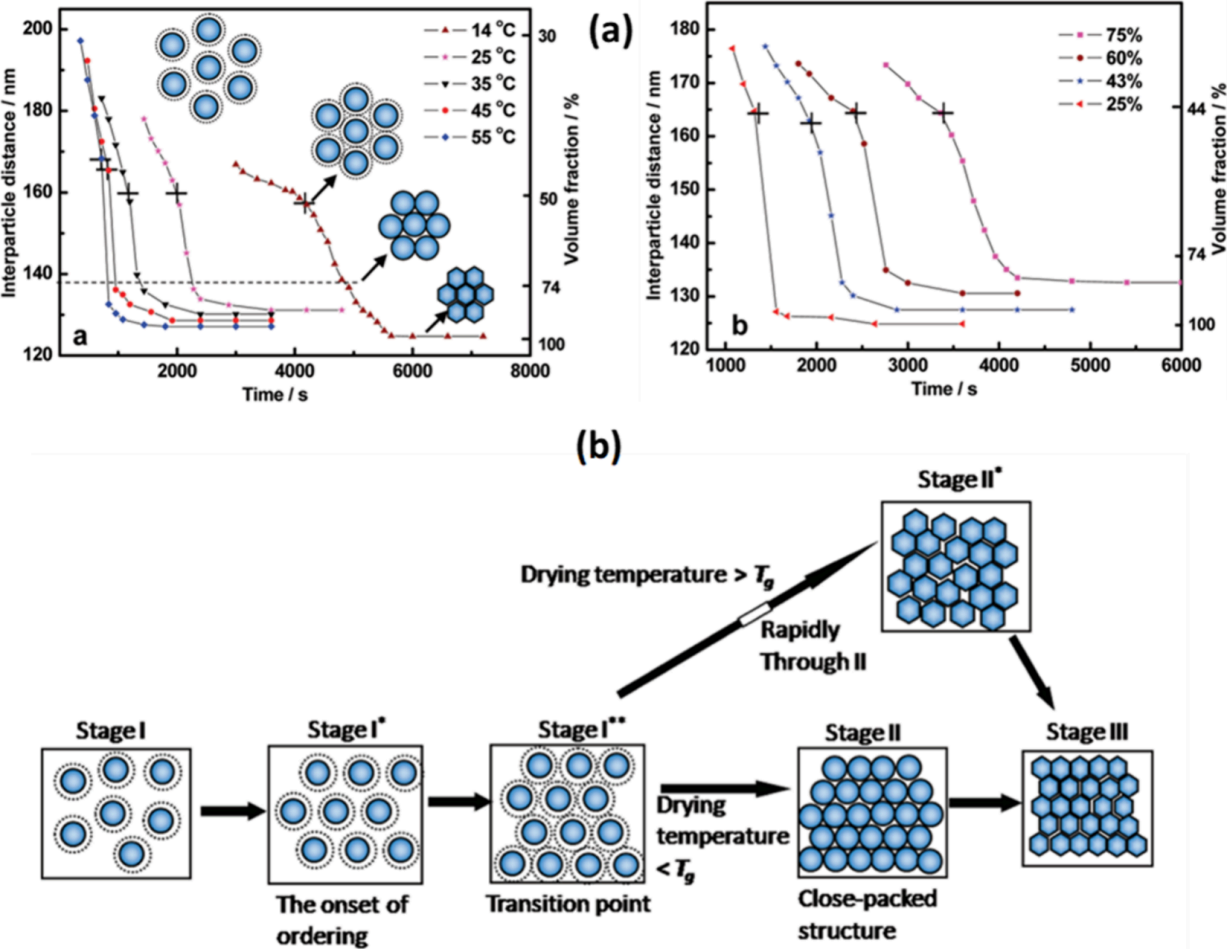
(a) The interparticle distance and volume fraction during film formation upon various environmental conditions, and (b) different stages of the proposed film formation mechanism. Reproduced with permission from ref (109). Copyright 2011 American Chemical Society.

The drying process was meticulously analyzed using TGA as depicted in Figure 14, highlighting a tripartite drying curve: a constant rate period, followed by an intermediate phase where the rate of evaporation slows due to irreversible particle interactions, and a final phase indicating further drying. Intriguing results showed that films dried at 14 °C retained less water, suggesting that lower drying temperatures might facilitate more efficient water removal, albeit with complex implications for film structure and particle coalescence. The residual water in the film was noted to escape through channels formed by nonpolymeric constituents, with these materials impeding complete particle coalescence at room temperature. Moreover, synchrotron SAXS analysis provided insights into the structural evolution of latex films, showing a transition from dispersed particles to a face-centered cubic crystalline structure upon drying. This crystalline arrangement was attributed to the presence of a protective membrane encapsulating the individual latex particles, which played a pivotal role in maintaining structural integrity. Temperature and humidity were found to modulate the degree of particle deformation, with films dried at 14 °C exhibiting complete deformation. Notably, a significant shift in volume fraction occurred as crystallization initiated, as depicted in Figure 15a. The shrinkage rate of the lattice, an essential parameter, responded dynamically to the evaporation rate. A notable acceleration in this rate marked a pivotal transition point, signifying further compaction and deformation of the latex films with prolonged drying. Ultimately, the study proposed a model for latex film formation that involves initial dispersion, colloidal crystallization at Stage I*, accelerated lattice shrinkage indicating particle compaction in Stage I**, and a final densely packed structure in Stage II, all illustrated in Figure 15b. This model underscores the complex interplay between drying conditions and film formation kinetics, offering a comprehensive framework for understanding the drying behavior of latex dispersions.

**Figure 16:**
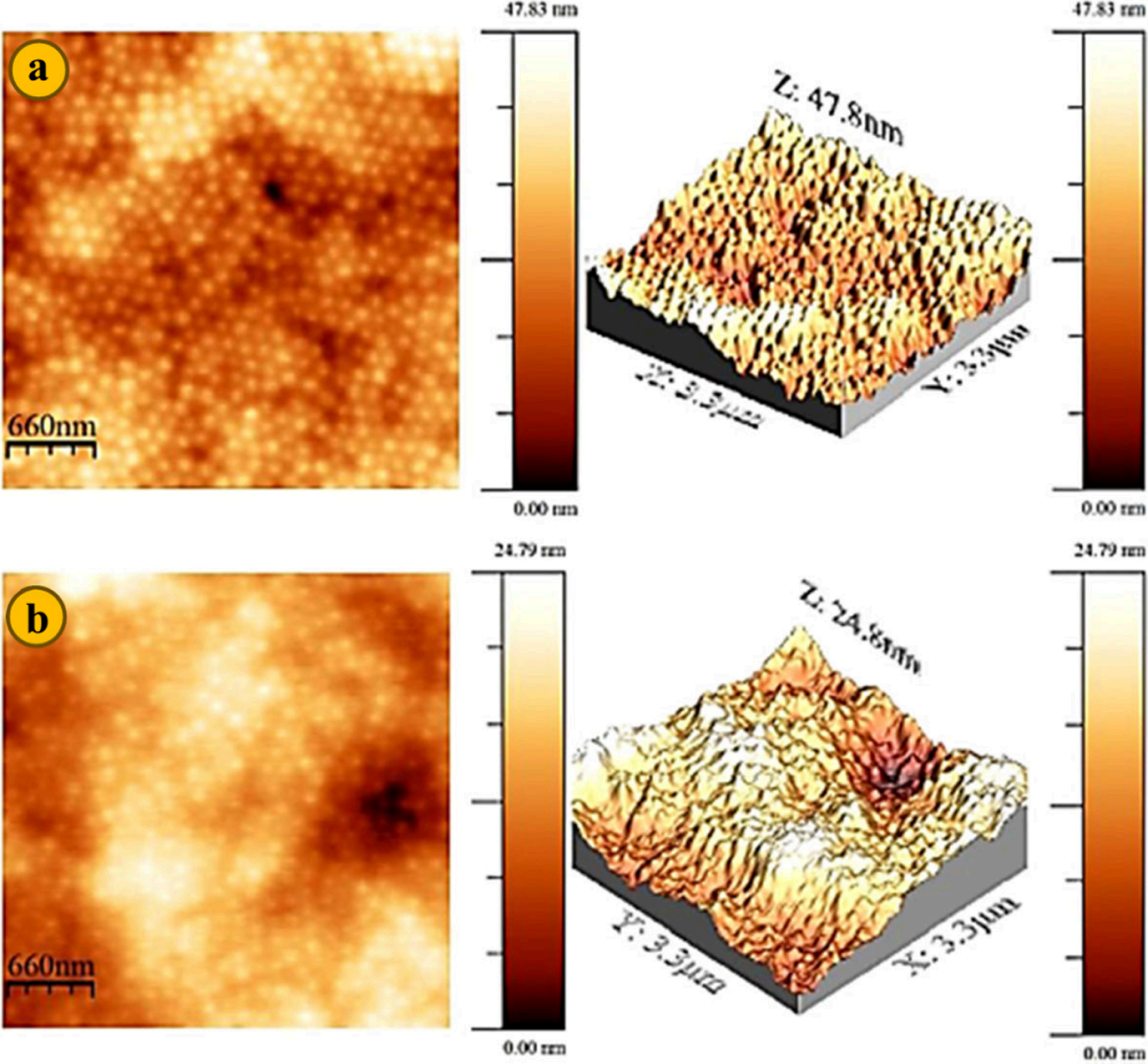
AFM height images of freshly applied (a) and cured films at 40 °C for 24 h (b). Reproduced with permission from ref (110). Copyright 2018 Shenyang Pharmaceutical University. Published by Elsevier.

Yang et al.’s research focused on the coalescence process of films from aqueous polymeric dispersions, using atomic force microscopy (AFM) to examine surface topography and nanoadhesion properties.^110^ Utilizing a nonionic copolymer of methyl methacrylate and ethyl acrylate with a *T*_g_ and MFFT of −8 and 5 °C, respectively, the study observed the effects of various curing conditions on film formation. Spin-coated films were subjected to ambient humidity conditions and cured at different temperatures (room temperature, 40 °C, and 60 °C) to simulate practical scenarios relevant to the pharmaceutical industry. AFM images (Figure 16) showed the evolution from freshly prepared films with closely packed colloidal particles, displaying a face-centered cubic ordering, to partially coalesced films after 24 h at 40 °C, indicated by a reduction in peak-to-valley distance from 47 to 24 nm. Further comparisons highlighted the impact of curing time and temperature on coalescence efficiency. Films left to cure at room temperature for 48 h resembled freshly prepared samples, while those cured at 40 °C for the same duration showed signs of cohesive film formation. A significant advancement in particle coalescence was noted in films cured at 60 °C, where the peak-to-valley roughness decreased substantially, emphasizing the importance of curing temperature in the film formation process. An experimental extension to assess the influence of humidity on film coalescence, curing films at 0% and 100% RH at room temperature for a month, revealed that colloidal particles remained visible with slight boundary-blurring, suggesting minimal coalescence under both dry and humid conditions. This observation, alongside RMS roughness measurements, indicated that temperature plays a more critical role than the humidity in the film-curing process, with higher temperatures significantly enhancing the coalescence rate and efficiency. Their findings underscore the complexity of the film formation process in aqueous polymeric dispersions, highlighting the significant role of curing temperature over humidity in achieving effective film coalescence. Despite varying humidity levels, temperature emerged as the dominant factor influencing the coalescence and surface roughness of the films. This study provides valuable insights into optimizing curing conditions to improve film formation, particularly emphasizing the need for controlled temperature settings to ensure cohesive and smooth films in practical applications, such as in the pharmaceutical industry.

Hall and colleagues conducted a comprehensive study on the influence of environmental conditions on the micromechanical properties of WB coatings.^111^ A seed latex, synthesized through seeded semibatch emulsion polymerization using a blend of styrene, butyl acrylate, and acrylic acid, resulted in a product with 37% solids content and 40 nm particle sizes, boasting a monomer conversion rate exceeding 99%.^112^ This latex formed the base for various coating formulations, some incorporating xanthan gum (XG) and poly(acrylic acid) (PAA) as dispersants. These formulations were applied to glass substrates and air-dried at 30 °C before being subjected to different relative humidity (RH) conditions. The study probed the interaction between water and the WB coating components, revealing that PAA experienced hydroplasticization, with a reduced *T*_g_ at higher RH levels.^113^ Conversely, XG absorbed water without a clear *T*_g_ change, and overall the thermal properties of the coatings were unaffected by RH. Mechanical analyses showed that the coating’s hardness and Hookean elastic modulus (EH) decreased under conditions of high RH and low temperature, particularly for the XG-laden samples, indicating hydroplasticization effects. Tack adhesion properties varied with RH and temperature; PAA low coatings showed increased tack adhesion with higher RH, while latex coatings experienced increased tack adhesion with rising temperatures above the *T*_g_, but decreased adhesion with higher RH at 30 °C. The presence of XG and PAA together suppressed tack adhesion. Viscosity, as analyzed by the Burgers model, generally decreased with rising temperatures and was variably affected by RH; for PAA low coatings, viscosity dropped with increased RH and temperature. Relaxation times (τ) are also inversely related to temperature and slightly lessened with higher RH. These results underline the complex dependence of WB coatings’ properties on both temperature and humidity, which has implications for their application and performance in various environmental conditions.

## 7 Practical Perspective and Concluding Remarks

### 7.1 The Imperative of Deep Understanding for Advanced Applications

The intricate process of film formation in WB coatings is not merely a subject of academic interest; it holds profound implications for the application efficacy and the longevity of protective layers in various industrial sectors. The final properties of a WB coating, including its durability, gloss, adhesion, and barrier properties, are directly related to how well the film forms. Poor film formation can lead to defects such as pinholes, cracking, or incomplete coverage, which compromise the coating’s protective function. By understanding the factors that influence film formation, formulators can adjust the composition of the coating to achieve the desired performance characteristics. This may involve modifying the polymer type, particle size, or the concentration of additives like surfactants, plasticizers, or coalescing agents. In addition, different application methods (e.g., spraying, brushing, and rolling) impose different stresses on the coating during film formation. Understanding how these methods interact with the film formation process is crucial for achieving consistent, high-quality results. For instance, spray application might require faster water evaporation and quick coalescence, while brush application may benefit from a slower process. WB coatings are favored for their low VOC content; however, ensuring that they perform as well as SB coatings requires a deep understanding of film formation. This understanding helps in developing formulations that meet environmental regulations without sacrificing quality.

#### 7.1.1 Application-Centric Film Formation Optimization

For application-centric optimization, the film formation process must be understood at a molecular level. This includes discerning how coalescence agents influence polymer interdiffusion or how surfactants and stabilizers modulate particle packing. Such knowledge enables the formulation of coatings with customized mechanical properties, adhesion qualities, and corrosion resistance, all of them critical for coatings tasked with protecting infrastructure in aggressive environments like marine or industrial settings. Even in two-component systems (e.g., epoxy-amine) with chemical curing through cross-linking reactions, the effectiveness of the chemical curing stage is strongly dependent on the quality of physical drying (film formation through evaporation, deformation, and then coalescence). Therefore, without a strong knowledge of all of the interactions and dynamic behavior of ingredients during film formation, it is not possible to improve this process in an efficient manner. As clear evidence, the emergence of coalescing agents in the WB coating technology, and then reaching to more and more efficient coalescing agents over decades would not be possible without knowing that the film formation is a result of the deformation and coalescence of polymer particles compressed to each other. With the continuous advancement of research instruments and technologies, it is possible to have better insights of molecular-scale mechanisms and probably revise the established knowledge in any field.

#### 7.1.2 Environmental Implications and Sustainability

The shift toward WB coatings is underpinned by an urgent need to mitigate the environmental impact of volatile organic compounds (VOCs) prevalent in SB coatings. Deep insights into WB film formation are essential to overcome the performance gap between WB and traditional coatings. By enhancing the water evaporation and particle consolidation phases, we can develop WB coatings that rival the protective qualities of SB alternatives without compromising environmental safety. Moreover, this understanding is crucial for reducing resource consumption and waste during the manufacturing process, thereby bolstering the sustainability profile of the coatings industry.

#### 7.1.3 Tailoring to Application Demands

In applications where durability under thermal, mechanical, or chemical stress is paramount, the ability to predict and control the film formation process translates to coatings that can withstand such conditions over extended periods. For instance, in the automotive industry, coatings must resist abrasion, maintain gloss, and prevent corrosion over the vehicle’s lifespan. Similarly, in the aerospace industry, coatings are expected to endure extreme temperatures and velocities. A profound comprehension of film formation empowers formulators to create coatings that meet these rigorous demands while maintaining environmental stewardship. Due to the complex nature of WB film formation as a result of the heterogeneous film formation behavior in both vertical and horizontal directions, high sensitivity to the environmental factors, and probably high sensitivity to the application conditions (e.g., substrate characteristics), thinking about different film formation mechanisms than a universal one looks more logical. The well-known three-stage film formation mechanism is only true in theory and ideal conditions, which is not the case in practical applications. Maybe the long-lasting failure of WB coatings to replace their SB counterparts for heavy duty applications, such as anticorrosive functionality under extremely corrosive environments, is due to the insufficient knowledge of their film formation nature leading to inefficient solutions.

### 7.2 Future Perspectives and Concluding Remarks

The field of WB coatings is on the cusp of transformative developments that are likely to redefine the future of surface protection technologies. As the world pivots toward sustainable practices, the importance of advancing our understanding of film formation mechanisms cannot be overstated. In particular, the quest to optimize these coatings for high-performance applications, such as in extreme environments, demands a nuanced appreciation of the intricate interplay between formulation chemistry, environmental factors, and application-specific requirements. This concluding section will delve into the future prospects of WB coatings, emphasizing the need for deeper insights into film formation, the critical role of sustainability, and the development of advanced anticorrosive functionalities suited for high and extreme conditions.

One of the most promising avenues for future research lies in the detailed study of film formation processes at a molecular level. While significant strides have been made in understanding the dynamics of particle packing, deformation, and coalescence, there remains much to be explored, particularly in the context of heterogeneous and complex formulations. The future will likely see the integration of advanced characterization techniques, such as atomic force microscopy (AFM) and in situ spectroscopic methods, with computational modeling to provide a more comprehensive picture of film formation. Such an integrated approach will allow for the precise control and prediction of film properties, enabling the development of tailor-made coatings that meet specific industrial demands. Moreover, the advent of machine learning and artificial intelligence (AI) offers new tools for deciphering the complexities of film formation. By analyzing vast data sets generated from experimental studies, AI can help identify patterns and correlations that may not be immediately apparent, leading to the discovery of novel formulation strategies. This data-driven approach could significantly shorten the development cycle of new coatings, allowing for the rapid prototyping and optimization of formulations with enhanced performance characteristics. In high-performance applications, particularly those involving extreme conditions such as high temperatures, aggressive chemicals, or intense mechanical stress, the integrity and durability of the coating film are paramount. Future research will need to focus on understanding how different environmental parameters, such as humidity, temperature, and UV exposure, influence film formation and long-term performance. By elucidating these relationships, it will be possible to design coatings that not only form robust films under challenging conditions but also maintain their protective properties over extended periods.

As environmental regulations become increasingly stringent, the demand for sustainable coating technologies is set to rise. WB coatings, by virtue of their reduced volatile organic compound (VOC) content, are inherently more environmentally friendly than their solvent-borne counterparts. However, the push toward sustainability extends beyond simply reducing VOCs. The future will require coatings that are produced from renewable resources, have minimal environmental impact during application, and can be safely disposed of or recycled at the end of their life cycle. In this context, the development of biobased polymers and additives for WB coatings represents a significant area of research. These materials, derived from renewable sources such as plant oils or lignocellulosic biomass, offer the potential to reduce the carbon footprint of coatings while also providing unique functional properties. For instance, biobased coalescing agents could be designed to enhance film formation without relying on traditional petrochemical-derived solvents, thereby reducing reliance on nonrenewable resources. Another critical aspect of sustainability is the reduction of energy consumption during the curing process. Traditional WB coatings often require elevated temperatures to achieve full film formation, which can be energy-intensive. Future coatings will need to be designed with low-temperature curing capabilities, potentially through the use of innovative catalysts or by exploiting the latent heat of environmental conditions. Additionally, the development of self-healing coatings that can autonomously repair minor damage could extend the service life of coatings, reducing the frequency of reapplication and thereby lowering the overall environmental impact.

The protection of substrates in harsh and extreme environments, such as offshore platforms, chemical plants, and aerospace applications, remains one of the most challenging and critical aspects of coating technology. In these settings, WB coatings must not only provide an effective barrier against corrosion but also withstand a range of physical and chemical stresses without compromising their protective capabilities. Future WB coatings will likely incorporate advanced nanomaterials and smart additives to enhance their anticorrosive properties. Nanoparticles, for example, can be used to create densely packed barrier layers that are impervious to water and oxygen, two primary drivers of corrosion. Additionally, the incorporation of corrosion inhibitors that are released in response to specific triggers, such as changes in pH or the presence of chloride ions, could provide targeted protection exactly when and where it is needed. The development of multifunctional coatings that combine anticorrosive protection with other desirable properties, such as hydrophobicity, antimicrobial activity, or thermal insulation, is another promising area of research. These coatings could offer comprehensive protection in environments where multiple forms of degradation occur simultaneously. For instance, in marine environments, a coating that resists both corrosion and biofouling could significantly extend the service life of structures such as ships or offshore wind turbines. Furthermore, as industries increasingly operate in extreme conditions, such as deep-sea exploration or high-altitude aviation, the thermal stability of coatings becomes a critical consideration. Future WB coatings will need to be engineered to maintain their structural integrity and protective properties across a wide temperature range. This could be achieved through the use of high-performance polymers that are resistant to thermal degradation, or through the incorporation of phase-change materials that help regulate the temperature at the coating surface.

The future of WB coatings lies in the adoption of a holistic approach to coating design, where the interplay between formulation chemistry, film formation processes, and application-specific requirements is fully understood and leveraged. This approach will necessitate close collaboration between chemists, materials scientists, and application engineers, as well as the use of advanced analytical tools and computational models. As the industry moves toward this integrated model, there will be a growing emphasis on the development of coatings that are not only effective and durable but also sustainable and environmentally responsible. The challenge will be to balance these often-competing demands, optimizing formulations to meet the rigorous standards of modern industrial applications while also minimizing their environmental footprint. In conclusion, the future of WB coatings is bright, with numerous opportunities for innovation and improvement. By deepening our understanding of film formation mechanisms, embracing sustainability as a core design principle, and developing advanced anticorrosive functionalities, the coatings industry can meet the challenges of tomorrow. As we look ahead, it is clear that the next generation of WB coatings will be more than just a protective layer; they will be smart, sustainable, and tailored to the specific needs of the most demanding applications.

## List of Abbreviations

WB: waterborne
SB: solvent-based
VOC: volatile organic compound
*T*_g_: glass transition temperature
MMA: methyl methacrylate
BA: butyl acrylate
EIS: electrochemical impedance spectroscopy
MFFT: minimum film formation temperature
SEM: scanning electron microscopy
wet STEM: wet scanning transmission electron microscopy
ESEM: environmental SEM
cryo-SEM: cryogenic SEM
TEM: transmission electron microscopy
MS-DWS: multispeckle diffusing wave spectroscopy
NMR: nuclear magnetic resonance
AFM: atomic force microscopy
EFM: electric force microscopy
SEPM: scanning electric potential microscopy
SANS: small-angle neutron scattering
SNOM: scanning near-field optical microscopy
FRET: fluorescence resonance energy transfer
BTA: benzotriazole
CPE: constant phase element
TPM: 2,2,4-trimethyl-1,3-pentanediol monoisobutyrate
RT: room temperature
RH: relative humidity
BG: 2-butoxyethanol
PnB: 1-butoxy-2-propanol
DPnB: di(propylene glycol) butyl ether
σ_c_: Warburg coefficient
PS-*co*-BA: styrene/*n*-butyl acrylate copolymer
SAXS: small-angle X-ray scattering
TGA: thermogravimetric analysis
XG: xanthan gum
PAA: poly(acrylic acid)
EH: Hookean elastic modulus
ηN: Newtonian viscosity
OCT: optical coherence tomography
